# To Pass or Not to Pass: Modeling the Movement and Affordance Dynamics of a Pick and Place Task

**DOI:** 10.3389/fpsyg.2017.01061

**Published:** 2017-06-28

**Authors:** Maurice Lamb, Rachel W. Kallen, Steven J. Harrison, Mario Di Bernardo, Ali Minai, Michael J. Richardson

**Affiliations:** ^1^Center for Cognition, Action and Perception, University of CincinnatiCincinnati, OH, United States; ^2^Department of Kinesiology, University of ConnecticutConnecticut, CT, United States; ^3^Department of Electrical Engineering and Information Technology, University of Naples Federico IINaples, Italy; ^4^Department of Engineering Mathematics, University of BristolBristol, United Kingdom; ^5^Department of Electrical Engineering and Computing Science, University of CincinnatiCincinnati, OH, United States

**Keywords:** behavioral dynamics, affordance dynamics, joint-action, pick and place, dynamical systems theory

## Abstract

Humans commonly engage in tasks that require or are made more efficient by coordinating with other humans. In this paper we introduce a task dynamics approach for modeling multi-agent interaction and decision making in a pick and place task where an agent must move an object from one location to another and decide whether to act alone or with a partner. Our aims were to identify and model (1) the *affordance* related dynamics that define an actor's choice to move an object alone or to pass it to their co-actor and (2) the trajectory dynamics of an actor's hand movements when moving to grasp, relocate, or pass the object. Using a virtual reality pick and place task, we demonstrate that both the decision to pass or not pass an object and the movement trajectories of the participants can be characterized in terms of a behavioral dynamics model. Simulations suggest that the proposed behavioral dynamics model exhibits features observed in human participants including hysteresis in decision making, non-straight line trajectories, and non-constant velocity profiles. The proposed model highlights how the same low-dimensional behavioral dynamics can operate to constrain multiple (and often nested) levels of human activity and suggests that knowledge of *what, when, where and how* to move or act during pick and place behavior may be defined by these low dimensional task dynamics and, thus, can emerge spontaneously and in real-time with little a priori planning.

## Introduction

Living and working in shared spaces often requires that individuals coordinate their actions together to accomplish shared behavioral goals. From a busy family preparing for the day to a couple casually loading a dishwasher together after a dinner party, interpersonal coordination often results in tasks being achieved more quickly and efficiently. Indeed, the addition of other individuals within a task action space constructively increases the complexity of (sub-)task behaviors over time by creating new (and destroying old) opportunities for action. Previous attempts to understand how the behavioral order of such joint-action coordination emerges over time have largely focused on identifying the representational and neural structures that support successful joint-action, including social action understanding and the perception of others intentional states (e.g., Rizzolatti and Craighero, 2004; Newman-Norlund et al., 2007; Graf et al., 2009; Sebanz and Knoblich, 2009). Equally important, however, is identifying the dynamical processes or laws that not only operate to constrain what and when behavioral actions are afforded during joint-activity, but also naturally shape the movements patterns or trajectories employed in the actualization of task relevant action possibilities.

Interestingly, previous research investigating the dynamical processes of coordinated joint-action and multiagent activity has demonstrated that the behavioral order of such activity is often *self-organized* and *synergistic*, naturally emerging from the task-relevant physical, biomechanical, and informational couplings and constraints that exist between co-actors and within a joint-action task space (e.g., Schmidt et al., 1990, 2012; Schmidt and O'Brien, 1997; Marsh et al., 2006; Frank and Richardson, 2010; Richardson et al., 2010; Riley et al., 2011; Anderson et al., 2012; Richardson and Kallen, 2015; Washburn et al., 2015). In turn, a growing number of researchers have also argued that multiagent activity is best conceptualized as a complex dynamical system and, moreover, that the behavioral order of self-organized, synergistic multiagent coordination can be understood and modeled using low-dimensional task or behavioral dynamics principles (e.g., Schmidt et al., 1990, 1998; Warren, 2006; Lagarde, 2013; Dumas et al., 2014; Richardson and Kallen, 2015; Richardson et al., 2015).

Motivated by this latter claim, the objective of the current study was to identify and model the dynamics that are relevant to social and joint-action object moving and passing tasks. As an initial exploration of these dynamics, a relatively simple object pick and place task was employed, in which one actor had to move objects from one tabletop location to another either alone or by passing the object to another co-actor. Of particular concern was identifying and modeling the *affordance* related dynamics that defined an actor's choice to move an object alone or to pass it to their co-actor and the trajectory dynamics of an actor's hand movements when moving to grasp, relocate, or pass the object. With regard to the latter aim, we were interested in determining whether the simple *behavioral dynamics* model of route selection and locomotory path navigation previously developed by Fajen and Warren (2003, 2004; also see Warren, 2006; Warren and Fajen, 2008) could be successfully generalized to model the smaller scale hand movement trajectories that occur during object pick and place tasks. We were also interested in determining whether an actor's choice of pass/release location is modulated by the location of the intended target location and/or the location of a co-actors hand. Below, we briefly review research and theory most relevant to these issues, prior to further detailing the specifics of the current study and the hypotheses being investigated.

### Affordances and affordance dynamics

Affordances are opportunities for action within an agent-environment system (Gibson, 1979; Michaels and Carello, 1981; Shaw and Turvey, 1981; Turvey et al., 1981; Reed, 1996; Chemero, 2003). More specifically, affordances are lawful agent-environment action potentials that capture the complementary relation (the “fit”) between an agent and the environment. For instance, a surface of a given height affords climbing (or not) in relation to an individual's body height and leg length (Warren, 1984). When sitting, an object is reachable (or not) based on the distance of the object relative to the arm-torso extension capabilities of the reaching agent.

Of course, if a human agent is allowed to stand and walk then any object is reachable and affords grasping so long as its size and weight are within the strength and grasping capabilities of the agent concerned. In addition to standing and walking over to grasp an object, a human agent could also use a stick or a pole to move an object within reaching distance. Similarly, if another agent with sufficient lifting capabilities is standing closer to a goal object, the human agent who wishes to reach and grasp the object in question could always ask that other agent move the object to a location within their reach or simply pass it directly into their hand. The significance of these latter examples is that they highlight how affordances are not only defined in relation to the bodily capabilities of an individual agent, but are also defined in relation human-tool systems (Shaw et al., 1995; Smitsman, 1997; Bongers et al., 2004) and joint-action or multiagent systems (Stoffregen et al., 1999; Richardson et al., 2007, 2010). The significance of this is that extending or increasing the degrees-of-freedom of one's perceiving-acting system via the embodiment of tools and cooperative co-action not only increases the number of different ways in which a certain affordance can be actualized, but can also increase the number of action possibilities or affordances that are available within an agent-environment system. For instance, a nail only affords hammering for a hammer-hand system. A large sofa only affords lifting and moving for a two-or more-person system.

With regard to understanding the dynamics of human and multiagent coordination, affordance research has revealed that action- or body-scaled ratios that capture the intrinsic relation between action relevant properties of an agent or multiagent system, A, and an environmental surface or object, E, can be used to predict critical shifts in the perception and/or actualization of affordances (e.g., Warren, 1984; Mark, 1987; Warren and Whang, 1987; Kinsella-Shaw et al., 1992; Richardson et al., 2007). For example, individuals spontaneously transition from reaching by extending their arm, to reaching by bending at the hip and extending their arm, to reaching by bending from an upright posture while extending their arm at critical action-scaled (E/A) ratios characterizing relevant relations between object distance and height in terms of the agent-environment system (e.g., Carello et al., 1989; Mark et al., 1997). Similarly, individuals' exhibit abrupt transitions between one-hand and two-hand grasping, and between one-person and two-person grasping at critical object-size/hand-size and object-size/arm-span ratios, respectively; typically at an E/A ratio of 0.75 (e.g., van der Kamp et al., 1998; Richardson et al., 2007). Accordingly, E/A (where E is a measured action relevant environmental property and A is the measured action relevant property the agent) represents a generic control parameter that not only defines the afforded state(s) of an agent-environment system, but also characterizes the stability of the behavioral modes employed to actualize those afforded states (e.g., Warren, 1984; Mark et al., 1997).

With this control parameter in hand, subsequent research investigating the dynamics of affordance actualization has revealed that individuals do not always transition from one behavioral mode to another at the same critical E/A ratio (i.e., exhibit *critical point transitions*). Rather, individuals typically exhibit *hysteresis*, in that they transition between different affordance related behavioral modes at different E/A values depending on whether E/A is increased over time or decreased over time (e.g., Fitzpatrick et al., 1994; van der Kamp et al., 1998; Richardson et al., 2007). For instance, individuals transition between one-hand and two-hand, and between one-person and two-person grasping at a higher E/A ratios when object size is scaled from small to large (approximately 0.85) than when object size is scaled from large to small (approximately 0.65; e.g., van der Kamp et al., 1998; Richardson et al., 2007). The significance of hysteresis with regard to understanding the dynamics of human behavior is that it implies multi-stability (two or more states or modes of behavior are stable over a range of control parameter settings), as well as nonlinearity (e.g., Strogatz, 1994; Kelso, 1995; Richardson et al., 2014). As such, affordance transitions can be conceptualized as bifurcation events, with affordance dynamics modeled as a nonlinear dynamical system (e.g., Frank et al., 2009; Lopresti-Goodman et al., 2011; Harrison et al., 2016).

### Joint-action pick and place behavior

In its simplest form a pick and place task involves an individual picking up a specified object and moving that object to a specified location. Understanding the nested sequencing of sub-action movements entailed by such behavior is non-trivial, however, given the large number of redundant degrees-of-freedom of the human movement systems and the underdeterminacy in end-point trajectories and/or joint angle configurations that this redundancy creates. Accordingly, there has been an extensive amount of research on such behavior, including research on the relationship between movement time, velocity, distance, and target goal size, path or trajectory length minimization, end-state comfort dynamics, end-effector vs. limb-joint control, hand-eye coordination, and so on (e.g., Fitts, 1954; Flash and Hogan, 1985; MacKenzie et al., 1987; Dean and Brüwer, 1994, 1997; Wolpert, 1997; Flash and Sejnowski, 2001; Jax et al., 2007; Rosenbaum et al., 2012). Of particular relevance here, is the well-established finding that given an obstacle free environment, humans tend to reach for and move hand-held objects along (i) a relatively straight line trajectory between pickup and drop-off locations, with (ii) a non-stationary, bell shaped, velocity profile that minimizes jerk and has a peak velocity between a 1/3 and 1/2 of the way through a movement (e.g., Fitts, 1954; MacKenzie et al., 1987; Dean and Brüwer, 1997; Flash and Sejnowski, 2001; Jax and Rosenbaum, 2007).

There is also a growing body of literature on joint-action pick and place behavior, including the effects of action observation on an actors' hand movement trajectories and grasping behavior (e.g., Becchio et al., 2008, 2012; Costantini et al., 2011; Ellis et al., 2013), the movement and action decision dynamics of individuals working independently of one another in a shared task space (Meulenbroek et al., 2007; Lorenz et al., 2014; Meyer et al., 2016; Scharoun et al., 2016), and when and how participants grasp, hold, and move objects together (e.g., Georgiou et al., 2007; Richardson et al., 2007; Vesper et al., 2009). As detailed above, joint-action pick and place behavior can also involve one agent passing an object to another agent when there is sufficient interaction between co-actors (Becchio et al., 2008; Meyer et al., 2013), with such interaction further increasing the constructive under-determinacy of how individuals are able to move an object from one location to another. Interestingly, although there is some recent evidence to suggest that individuals tend to pass objects to co-actors in a manner that maximizes the beginning-state comfort of the co-actor (so called, third–order motor planning; e.g., Ray and Welsh, 2011; Meyer et al., 2013), little is known about the location where actors choose to place or release an object for another co-actor in an under-constrained joint-action pick and place task. Indeed, when one actor chooses to pass, place, or release an object for another individual to move within a real-world context, a specific release/passing location is rarely pre-defined or specified prior to the passing action. A modest number of studies have started to examine this latter question within the context of human-robot interaction (e.g., Cakmak et al., 2011; Strabala et al., 2013) and have found that individuals prefer predictable pass locations and orientations. However, the highly constrained nature of the task contexts and object hand-over manipulations employed in these latter studies means that it is hard to generalize the results of these studies to human-human pick and place behavior (also see Shibata et al., 1995). Accordingly, a sub-aim of the current study was to begin to address this gap in the literature and, in particular, begin to identify the degree to which individuals spontaneously choose object pass and release locations as a function of a waiting co-actors hand location and/or the final target goal location of the to-be-moved object.

Both the previous research outlined in this section and our own piloting indicated that when neither co-actor was constrained, it was not clear whether pass decisions and locations depended on co-actor movements while awaiting a pass, co-actor movements once they received a pass (i.e., the passer's perception of the receiver's action capabilities), or all decisions depended only on features of the task environment. Often the person waiting on the pass would move prior to receiving the pass, though what drove that movement was not clear from the data. Notably, this means that in order to interpret and model joint-action pick and place behavior, we first needed to model and understand features of pass decisions in a social pick and place task, where interactions between co-actors are minimized. Thus, while the current task involved social action it was not a joint-action task (Becchio et al., 2008, 2012). By starting with the current social action task, the results of the current experiment will facilitate understanding and modeling joint-action pick and place behaviors when pass decisions and behaviors are relatively unconstrainted and co-actor behaviors become more interdependent. As such, the task we present in this paper is important for joint-action research because it fills significant gaps in the literature on joint-action pick and place tasks, including understanding where and when individuals pass to a human co-actor in an otherwise unconstrained task space.

### Modeling behavioral dynamics

The term “behavioral dynamics” refers to a general framework for understanding and modeling the complex movement dynamics that characterize the behavior of actors within an agent-environment system. First detailed by Warren (2006) in order to understand the complex movement patterns of individuals performing solo-action tasks, the approach employs *task specific* models (Saltzman and Kelso, 1987) to discern the dynamics of coordinated behavior, and is equally applicable to joint-action and multiagent activity (e.g., Dachner and Warren, 2014; Rio and Warren, 2014; Richardson et al., 2015). Consistent with the more general dynamical and complex systems approach to human behavior (e.g., Kugler et al., 1980; Saltzman and Kelso, 1987; Thelen et al., 1994; Richardson et al., 2014), it places a strong emphasis on self-organization and contextual emergence, and, in turn, attempts to formally (mathematically) model human and multiagent behavior as emerging from the lawful interaction of physical and informational processes, biomechanical couplings, and contextual constraints.

A key requirement for modeling the behavioral dynamics of a specific action or movement task effectively is to define a functional, yet low-dimensional description of the corresponding task space. This includes appropriately defining (i) the task goal in terms of the relevant terminal objective, (ii) the minimal number of task dimensions (i.e., axes and task variables) required to express this terminal objective, and (iii) the task dynamic topology (equations of motion) for each task dimension and degree-of-freedom (Saltzman and Kelso, 1987; Warren, 2006). A foundational example of such task dynamics modeling is provided by the work of Fajen and Warren (Fajen and Warren, 2003, 2004; also see Warren, 2006; Warren and Fajen, 2008 for a review), in which the authors successfully modeled the self-organized behavioral dynamics of human locomotory navigation and route selection. Although the complete model proposed by Warren and Fajen is able to successfully capture route switching dynamics in relation to moving and stationary environmental goal locations and obstacles, of primary relevance here is the simple manner by which they modeled the locomotory trajectories of agents moving from an arbitrary start location to a fixed goal position. In this (sub)model, a locomoting agent was defined abstractly (at the whole-body level) as a directional point-mass within a Euclidian (*x, y*) planar task environment, with the agent's heading direction, φ, and the angle of the target goal location, θ_*g*_, defined with respect to one of the planar task axes (i.e., an exocentric reference frame was employed). The terminal objective of the locomoting agent was then defined as simply turning toward a target goal location by changing their heading direction or turning rate, $\dot{\varphi }$, until φ−θ_*g*_ = 0. The topology of this terminal objective was captured using the adapted mass-spring system.

(1) $$\ddot{\varphi }=-b_{g}\dot{\varphi }-k_{g}\left(\varphi -\theta_{g}\right)f(d_{g}),$$

where $\dot{\varphi }$, and $\ddot{\varphi }$, corresponds to the velocity and acceleration of the agent's heading angle, φ, and *b* and *k* are damping and spring/stiffness terms, such that ${-b}_{g}\dot{\varphi }$ acts as a friction force on the turning rate, and the function −*k*_*g*_(φ − θ_*g*_) operates to minimize the difference between the agent's current heading angle, φ, and the angle, θ_*g*_, that will lead the agent toward the goal. Finally, *f*(*d*_*g*_) is a function that modulates the rate of change in heading angle as a function of the distance, *d*_*g*_, to the goal—typically this is set such that the closer the goal the more rapid deviations of φ away from θ_*g*_ are minimized.

Although it might be hard to imagine that a simple system such as Equation (1) could effectively capture any form of complex human movement behavior, the ability of Equation (1) to successfully predict the steering and locomotory navigation behavior of human agents has been verified across numerous experimental procedures and environmental task contexts and with the addition of a similar obstacle avoidance function^1^ the model has provided strong evidence that such behavior can emerge without a priori planning as a self-organized result of interacting environmental attractors and repellers (see Warren, 2006; Warren and Fajen, 2008 for reviews). Recent research has also demonstrated how similar route selection equations can be extended to a range of complex multi-agent locomotion or pedestrian tasks (e.g., Dachner and Warren, 2014; Rio and Warren, 2014) and that the behavioral dynamics approach more generally can be employed to understand and identify the low-dimensional dynamics laws the underlie a wide range of joint-action and multiagent movement coordination tasks (e.g., Lucas et al., 2015; Richardson et al., 2015, 2016).

### Current study

As stated above, the objective of the current study was to begin to explore the behavioral dynamics that underlie social and joint-action object moving and passing tasks using a relatively simple object pick and place task, in which one participant had to move objects from one tabletop location to another either alone or by passing the object to a co-actor. The key manipulation was the relative distance of the starting (appearance) and target goal (drop-off) locations of the to-be-moved object with respect to the standing position of the participant and co-actor, with a specific range of appearance and drop-off locations chosen to identify and model three central facets of social and joint-action pick and place behaviors, namely: (1) the affordance dynamics that characterized an actor's choice to move an object alone (i.e., not passing) or passing it to the co-actor; (2) where an participant chooses to pass/release an object and the degree to which this pass location is modulated by the location of the intended target location; and (3) the trajectory dynamics of the participant's hand movements when moving toward, with, or passing an object.

Based on the previous research outlined above, we expected that participants would transition between passing and not-passing behavior as a function of their arm/torso reach capabilities. Of more interest, was determining what environmental variables operated to define the corresponding E/A control parameter. For the current task, we expected that target location would largely moderate a participant's passing decision. However, it was possible that an object's appearance distance might also operate to constrain passing decisions. We also expected that participants would exhibit hysteresis when the relative distance of the target location from the grasping agent was increased vs. decreased over time, indicative of a multistable, nonlinear dynamical process that could be modeled accordingly.

We had no a priori predictions with regard to the location that participants would choose to pass/release objects for their co-actor to pick up given the lack of previous research on this question. In general, however, we did expect that participants would exhibit a stationary and highly predictable pattern of behavior (Shibata et al., 1995; Cakmak et al., 2011; Strabala et al., 2013), either choosing a single pass/release location or passing/releasing objects in a position functionally related to the intended target location and the co-actors hand position.

With regard to the hand-movement trajectories of participants, we expected that the spatial dynamics of these movements would be qualitatively similar to the goal directed locomotory movements observed by Fajen and Warren (2003, 2004) and, thus, could be model using a adapted (extended) version of Equation (1). Note, however, that in contrast to the constant velocity assumption underlying the Fajen and Warren behavioral dynamics model of locomotory movements, we expected participant movements to exhibit a non-constant velocity profile and that a corresponding non-constant velocity function would need to be developed in order to successfully model the pick and place movements investigated here.

## Materials and methods

### Participants

Sixteen University of Cincinnati students (aged 18–28 years) were recruited to participate in the experiment. 8 male and 8 female participants took part in the study. Participants received credit as a part of a class requirement for an undergraduate Psychology course. All participants provided written consent prior to completing the study, with the procedures and methodology employed reviewed and approved by the University of Cincinnati Institutional Review Board.

### Materials and apparatus

**Figure 1:**
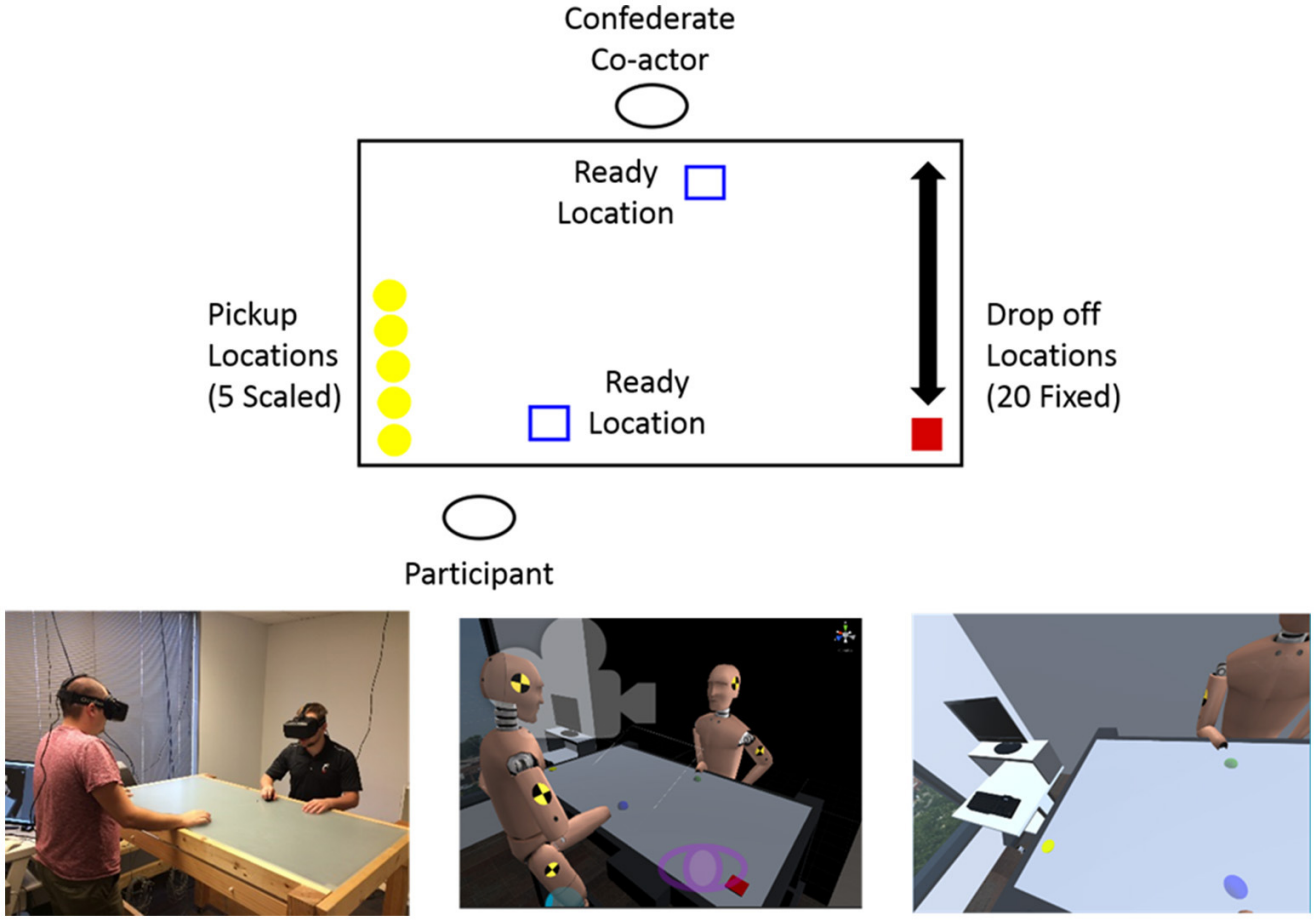
**(Top)** Illustration of experimental setup. Before a trial began only the blue ready squares appeared on the table. When the trial began one yellow disc appeared on the participant's left and one red target square appeared on their right. (**Bottom left**) The experimental room set up. (**Bottom Center**) The virtual environment and avatars at the beginning of a trial. (**Bottom right**) The view of the participant at the beginning of a trial as seen in the head mounted display.

An illustration of the experimental task setup is displayed in Figure 1. As can be seen from an inspection of this figure, the participant and co-actor stood in front of 1.5 × 0.89 × 1.15 m table in a 3 × 4.9 m laboratory room and completed the object moving and passing task in a room-scaled virtual environment in which the virtual laboratory and table were isomorphic in size and location. The co-actor (henceforth confederate co-actor) in this experiment is a lab assistant and is known to the participant to be a lab member. The physical table acted as a solid surface that both limited the participant and confederate co-actor movements within the virtual environment and created a surface on which the participant and confederate co-actor could move a hand-held wireless Polhemus Latus motion-sensor (Polhemus Ltd, Vermont, USA) that tracked their right hand movements within the virtual environment at 96 Hz. The participant was positioned on one side of the table, standing half way between the middle of the table and the pickup location, with the confederate co-actor positioned in the middle of the table on the opposite side.

The virtual environment, task objects, and task controllers were designed using the Unity 3D game engine (version 5.2.0; Unity Technologies, San Francisco, California) and Sketchup 2015 (Tremble Navigation Technologies, Sunnyvale, California). The virtual environment and task objects were presented to participants using an Oculus Rift DK2 headset (Oculus VR, Irvine, California), which had a vertical field of view of 105° and a horizontal field of view of 94°. The participant and confederate co-actor's head movements were also tracked using Oculus Rift DK2 head tracking system. Separate computers connected by a LAN connection powered the Oculus Rift DK2 HMDs, with each computer handling the rendering of the virtual environment and controlling the head movements for the participant and confederate co-actor. The Host computer (participant) handled the motion tracking inputs, task controllers, and data recording. The maximum display latency between the participant and confederate co-actor real-world movements and their movements in the virtual environment was 33 ms. The experimental task states, including positions of participant and confederate co-actor's hands and head position, the appearance state and position of the target objects, and which individual was in possession of a target object, were continuously recorded at 70 Hz.

Virtual reality was employed for the current experiment because it offered two immediate advantages over a real world pick and place task: (1) the task reset time between trials can be instantaneous when using virtual reality allowing for a large number of trials to be completed in a timely manner and (2) the virtual environment allows for improved control of possible confounds, limiting visual task and behavioral information available to each co-actor to only that which is being explicitly tracked during the task, i.e., task states, right hand movements, and head movements. Moreover, as a future goal of this line of research is implementation of the proposed dynamical model in artificial agents, the virtual reality paradigm provides an ideal apparatus for obscuring the identity/origin of co-actor behaviors.

Within the virtual environment, the participant and confederate co-actor were represented as identical virtual avatars modeled after a crash test dummy with a height of 1.8 m, with the virtual environment being identical for the participant and confederate co-actor except for the fact that they were positioned on the opposite sides of the virtual table. The height of the participant and confederate co-actor's visual field was also calibrated such that their viewing height was equivalent regardless of their actual height. Both the participant and confederate co-actor's right hands were represented by a semi-transparent blue sphere at the end of the dummy's right wrist in order to simplify interaction with the task environment. The participant and confederate co-actor's hand-held wireless Polhemus Latus motion-sensors controlled the movements of this sphere. An inverse kinematics controller (model and controller supplied by Root Motion, Tartu, Estonia) driven by these motion sensor movements and the head movements of the participant and confederate co-actor controlled the right arm and body movements of the participant and confederate co-actor's virtual avatar, respectively. The resulting arm and body movements were not identical to the real world arm and body movements of the participant and confederate co-actor, but were close enough to render any differences between the real and virtual body postures of the participant and confederate co-actor unnoticeable or not functionally relevant.

### Experimental task

The experimental task required a participant to move virtual disc objects that appeared on one side of the virtual tabletop to an indicated target location on the opposite side of the virtual table, with a choice of either moving the object alone or passing the object to the confederate co-actor. The disc objects always appeared on the participant's left side and the target location, specified by a red square, always appeared on the participant's right. A trial began when both the participant and confederate co-actor indicated they were ready by placing their sphere/hand in a blue ready location (blue square) displayed directly in front of them on the virtual table. When both the participant and confederate co-actor's virtual hands were ready, the ready locations would disappear and a disc would appear in one of 5 pickup locations along with one of 20 red target locations. The participant was instructed to pick up the disc when it appeared and attempt to move it to the target location. A pickup occurred when the participant's sphere came in contact with the disc. When picked up, the disc moved with the participant's hand until it reached the target or the participant passed the disc. The participant was informed that if the reach to the target was either too far or uncomfortable, they could pass it to the confederate co-actor. A pass involved picking up the disc and then releasing it somewhere on the table by lifting their hand from the table. Importantly, the confederate co-actor was instructed to remain at the ready position unless the participant initiated a pass (i.e., they were instructed not to move prior to a participant initiating a pass by releasing the object for them to pick up). This instruction insures that pass decisions and locations are not influenced by anticipatory or communicative movements initiated by the pass receiver. While such movements may be important to more complicated pick-and-place task, they can obscure how task features and passer preference affect pass behavior^2^. To complete a pass, the confederate co-actor would pick up the disc and move it to the target. A trial was completed when the disc reached the target. Upon trial completion the disc and target would disappear and the ready boxes for the next trial would appear. The participant's preferred reach was recorded before completing the experiment by asking the participant (inside the virtual environment) to reach to the farthest comfortable point along a blue line that appeared along the left side of the table. This reach distance was then used to scale the 5 appearance pickup locations to each participant's preferred reach distance. The 5 disc appearance/pickup positions, illustrated as yellow circles in Figure 1, were located along the same axis as the calibration line on the table extending perpendicular to the participant. These appearance/pickup locations corresponded to 20, 40, 60, 80, and 100% of a participant's preferred reach distance (i.e., E/A ratios of 0.2, 0.4, 0.6, 0.8, and 1.0)—the average reach distance of participants was 52.2 cm (*SD* = 6.98 cm). Relative to the ready/start location these object pickup locations were positioned at a negative x-distance of 32.3 cm and had mean y-positions of −1.4, 7.2, 15.8, 24.4, and 33 cm, respectively.

The same 20 unscaled target locations on the right side of the table were used for all participants. These target locations were equally spaced from the near to the far edge of the participants' side of the table. Relative to the start/ready location these had positive x-distance of 103.7 cm and y-positions from −7 cm to 59.5 cm in 3.5 cm steps.

### Procedure

Participants were told that the experiment was investigating the dynamics of object pick and place behavior and that they would be completing a simple pick and place task with a confederate co-actor. The participants and confederate co-actor were then embedded within the virtual environment using the HMD and viewing height, sensor, and appearance location calibration was performed. Task instructions were then provided to the participant and after participants indicated that they understood the task procedure and goal, experimental trials began. Participants were told that the task would involve 600 trials and that if the reach to the target was either too far or uncomfortable, they could pass the object to the confederate co-actor. Moreover, participants were encouraged not to strain themselves in order to reach a target. No further instructions regarding when or where to pass were given to participants.

Experimental trials were broken up into 3 blocks of 200 trials (i.e., 5 appearance/pickup locations × 20 target locations × 2 trials for each appearance-target location combination). In the first and third blocks of trials, the discs appeared sequentially, either progressively moving away from the participant (ascending) or toward the participant (descending) over trials with appearance order counterbalanced across participants. During these blocks, each pickup/appearance location was presented 40 times in a row with each presentation occurring twice for each of the 20 target locations, once while target locations appeared in an ascending order and once when they appeared descending order. Participants always experienced the same ascending-descending or descending-ascending order across appearance locations in the first and third trial blocks, with these target appearance conditions counterbalanced across participants. In the second block of trials, each pickup-target location pair was presented twice in a random order from trial to trial. After each 200 trial block, the participant and confederate co-actor were given an opportunity to rest before continuing to the next block. Blocks lasted between 10 and 15 min.

## Results and discussion

The current pick-and-place task was designed to address three related questions. First, what task variables determined the participants' decision to pass or not pass an object and what were the associated affordance related dynamics of these behavioral events? Second, where did participants choose to release the object when passing the object to a co-actor and to what degree was the pass location functionally related to the intended object goal location and/or the confederate co-actors hand location. Third, what were the trajectory dynamics of the participant's hand movements when moving to grasp, relocate, or pass an object within a two-dimensional task space. Below we consider each of these questions in turn.

### What drove pass decisions?

For the pick and place task investigated here, there were essentially two relevant distance-related task variables that were likely to have influenced the participant's pass/no-pass behavior: the distance from the participant's ready location to the object pickup location and the distance from the participant's ready location to the object target drop-off location. Note that, by instructing the confederate co-actor to passively wait for passes, we have effectively eliminated the possible complicating (but potentially important) role anticipatory or communicative movements on behalf of the pass receiver. As a preliminary examination of the relationship between these two task variables and the participants' dichotomous, pass or not pass decisions, separate point-bi-serial correlations were conducted on the trial-by-trial pass/no-pass data series for each participant for each trial block (i.e., ascending-descending, random, and descending-ascending target location trial blocks). As can be seen from an inspection of Table 1, only target location was significantly correlated with the participant's pass/no-pass behavior across trials, with an overall average correlation between the participant's trial-by-trail pass/no-pass behavior and target location of 0.796 (*SD* = 0.074; *p* < 0.001)^3^. In other words, the distance of the object pickup location appeared to have no effect of pass/no-pass behavior, with pass/no-pass behavior almost completely driven by the distance of target goal location^4^.

**Table 1:** Average point-bi-serial correlations for participant's trial pass decision.

		**Pickup locations**	**Target locations**
Block 1 (Ascending-Descending)	r_pb_	−0.031	0.817
	(SD)	(0.093)	(0.040)
	p	0.444	0.000
Block 2 (Random)	r_pb_	0.001	0.782
	(SD)	(0.022)	(0.100)
	p	0.856	0.000
Block 3 (Descending- Ascending)	r_pb_	0.009	0.791
	(SD)	(0.045)	(0.081)
	p	0.601	0.000

**Figure 2:**
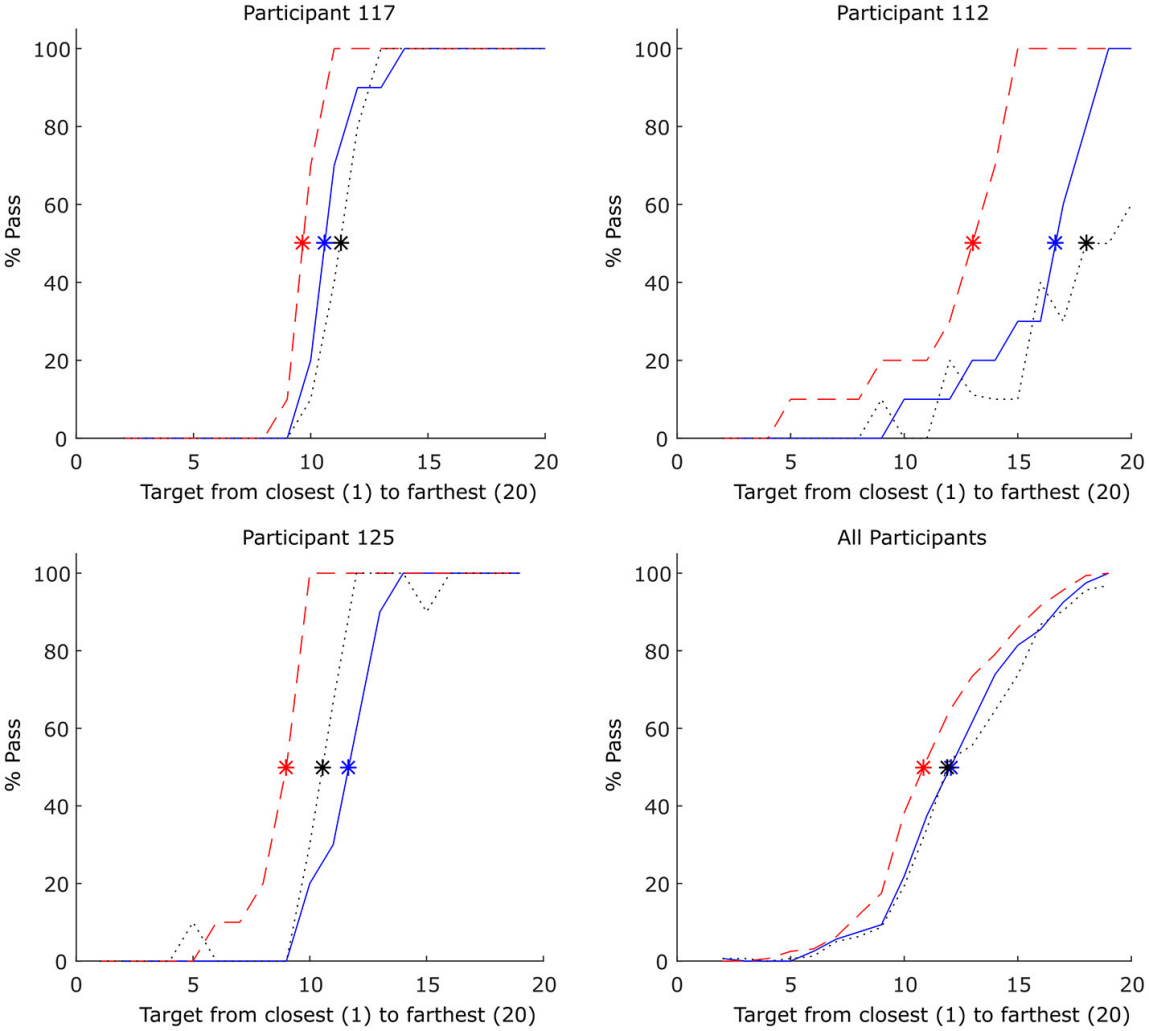
Percent passes for each target location (*n* = 20) for 3 example participants (**Top left** and **right** and **Bottom left**) and averaged over all participants **(Bottom right)**. The red dashed line indicates the percentage of passes for each target as the targets are moving away from the participant. The blue solid line indicates the percentage of passes as the targets are moving toward participant. Random target appearance order is represented by the black dotted line. Asterisks represent the point at which 50% of decisions were passes and 50% were not (note that this point could occur between target locations). Each target location was presented 5 times each per Ascending and Descending conditions and 10 times for the Random condition.

With regard to the target distance that participants transitioned between passing and moving the object alone, this occurred at an average y-target distance of 42.4 cm (*SD* = 9.17), which corresponded to an E/A ratio (i.e., y-target-distance/participant comfort reach distance) of 0.823 (*SD* = 0.19). Consistent with previous affordance research (e.g., Fitzpatrick et al., 1994; van der Kamp et al., 1998; Richardson et al., 2010), participants also exhibited hysteresis with the pass/no-pass transition occurring at an average E/A ratio of 0.853 (*SD* = 0.24; target y-distance of 43.7 cm) for the ascending target distances and 0.797 (*SD* = 0.21; target y-distance of 40.8 cm) for descending target distances, indicating that the relative stability of passing and non-passing behavior was more or less equivalent across this E/A parameter range (see Figure 2). To verify that this hysteretic effect was significant, a one-way repeated measures ANOVA comparing the distance (target location) that participants switched between passing and non-passing behaviors as a function of target location order (i.e., ascending, descending, and random), was conducted. Using a Greenhouse-Geisser correction this analysis revealed a significant effect of target location order, *F*_(1.44, 21.606)_ = 8.908, *p* = 0.003, $\eta_{p}^{2}\;$ = 0.373, with Bonferroni post hoc analysis indicating that pass/no-pass transition distance for the ascending target order was significantly higher compared to the pass/no-pass transition distance for the descending target order (*p* = 0.027). There was no difference between the ascending and random target location orders (*p* = 0.541), but there was a significant difference between descending from random location orders (*p* = 0.015).

### Where did participants release/pass objects?

A Pearson correlation analysis revealed that, for a majority of participants, the (*x, y*) tabletop location where they released (passed) objects for the confederate co-actor during passing trials was significantly correlated with (i) the pass location chosen on the previous passing trial, (ii) the target location, and (iii) to a much lesser extent, the object pickup location (see Table 2). Separate hierarchical linear regression analyses were conducted on each participant's passing trial event series as a function of trial block, with trial pass location as the dependent variable and location of the previous pass, target location, and pick-up location sequentially entered as independent variables. As can be seen from an inspection of Table 3, this analysis revealed that on any given passing trial a participant's previous object release/pass location was the dominant predictor of a participant's current object release/pass location, with current target location and pickup location only slightly increasing the percentage of variance accounted for. This suggests that participants tended to more or less pick a location to release/pass the object for the confederate co-actor during early passing trials and then stick with that location across passing trials. To further verify the latter possibility, a cluster analysis was conducted, using the K-means cluster analysis algorithm, which finds cluster centers that minimize the sum of squared error (SSE) for a given number of clusters, k. We analyzed the release/pass locations to determine whether these locations typically clustered around 1, 2, or 3 cluster centroids. The optimal number of clusters was defined as the value of k such that the difference of the SSE for a reference distribution, determined by Monte Carlo sampling of a reference distribution, was greatest compared to the other values of k.

**Table 2:** Average correlations between participants' trial-by-trial pass locations and object pickup and target locations, as well as participants previous pass decision, as a function of trial block.

		**Pickup locations**	**Target location**	**Pass decision previous trial**
Block 1	r	−0.153	0.462	0.541
	(SD)	(0.367)	(0.262)	(0.162)
	p	0.107	0.100	0.000
	(SD)	(0.186)	(0.259)	(0.001)
	% sig. < 0.05	68.75%	87.5%	100%
Block 2 (Random)	r	−0.098	0.344	0.243
	(SD)	(0.143)	(0.219)	(0.130
	p	0.451	0.167	0.167
	(SD)	(0.316)	(0.294)	(0.224)
	% sig. < 0.05	12.5%	68.75%	50%
Block 3	r	−0.038	0.172	0.405
	(SD)	(0.234)	(0.271)	(0.178)
	P	0.306	0.339	0.057
	(SD)	(0.336)	(0.357)	(0.124)
	% sig. < 0.05	50%	37.5%	81.25%

*% sig. < 0.05 equals to the percentage of participants who exhibited a significant relationship between pass location and the corresponding task variable*.

**Table 3:** Average hierarchical linear regression results for participants' trial-by-trial pass locations as a function of trial block.

		**Model 1 Location of previous pass**	**Model 2 Location of previous pass target location current trial**	**Model 3 Location of previous pass target location current trial pickup location**
Block 1 (Ascending-Descending)	r	0.583	0.640	0.678
	(SD)	(0.182)	(0.189)	(0.179)
	F	99.655	62.553	49.971
	p [% sig.]	0.000 [100%]	0.001 [100%]	0.002 [100%]
Block 2 (Random)	r	0.239	0.454	0.485
	(SD)	(0.125)	(0.167)	(0.164)
	F	6.214	18.380	13.869
	p [% sig.]	0.167 [50%]	0.062 [81%]	0.078 [81%]
Block 3 (Descending- Ascending)	r	0.405	0.442	0.467
	(SD)	(0.184)	(0.186)	(0.181)
	F	35.629	23.683	16.511
	p [% sig.]	0.057 [81%]	0.082 [75%]	0.113 [75%]

*[% sig.] equals to the percentage of participants who exhibited a significant relationship between pass location and the corresponding task variable*.

**Figure 3:**
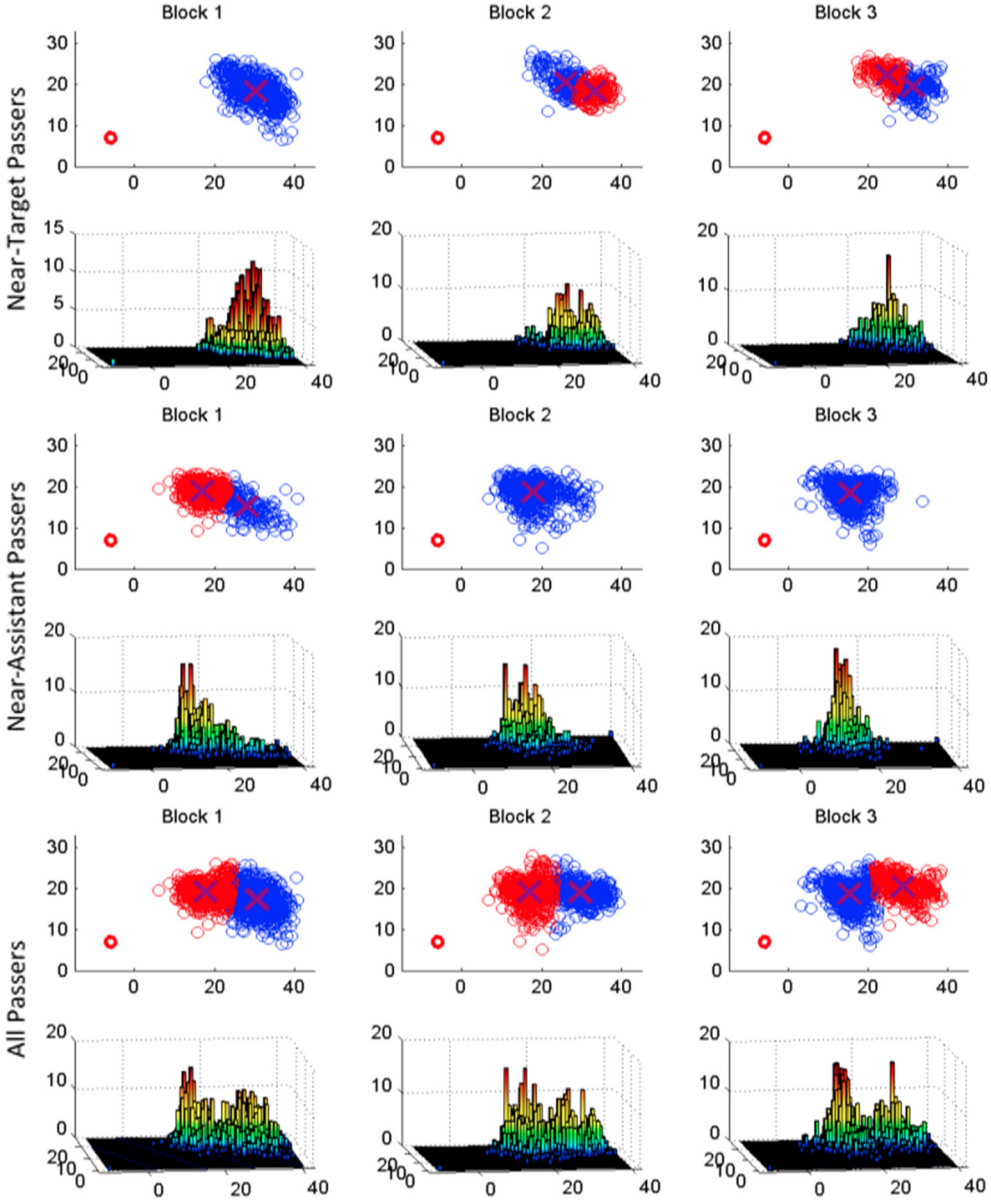
Pass locations split by pass location strategy. The top row of each pass strategy section provides a 2d plot of all pass locations for that strategy. The optimal number of clusters was calculated as above, using either 1 or 2 clusters, and k-means cluster analysis was performed. Conditions with more than 1 cluster have red and blue drop locations. The bottom plots in each section provide a 3-d histogram of the drop locations in order to illustrate frequency of drops in a given region and location. The red circle in the bottom right corner of each plot illustrates the size of the disc object.

The results of this K-means cluster analysis can be seen in Table 4. As expected given the preliminary correlation and regression analysis reported above, for the majority of participants the optimal number of clusters was 1 within the same trial block. However, as can be seen from an inspection of Figure 3, participants appeared to adopt one of two object release/pass location strategies. That is, release/pass locations tended to occur in one of two general areas of the task space, with some participants exhibiting a tendency to release/pass objects nearer to the confederate co-actor's hand, while other participants tended to release/pass the objects nearer to the object target (drop-off) locations. This is particularly clear from an inspection of the 3D histograms of all participant pass locations in the bottom panel of Figure 3, where two distinct peaks appear in the histograms corresponding to the two passing regions. Using k-means cluster analysis to define these 2 location clusters (i.e., specifying *k* = 2 clusters for all participant pass locations) we observed that 8 participants made more than 50% of their release/passes in the cluster region closest to the confederate co-actor's ready/start location (near-confederate co-actor region; see middle panel of Figure 3) and 6 participants made more than 50% of their release/passes in the cluster region closest to the targets (near-target region; see top panel of Figure 3). The remaining two participants began the experiment releasing/passing in the near-target region, but then in blocks 2 and 3 released/passed most of their passes in the near-assistant region. For those who always released/passed in the same region, the near-target participants (*n* = 6) released/passed objects in the near target region on average 94.8% of the time in the near-target region and the near-confederate co-actor participants (*n* = 8) released/passed objects in the near confederate co-actor region on average 89.1% of the time, further indicating that individuals tended to pick a general table location to pass/release objects for the confederate co-actor and then continue pass to that region across passing trials.

**Table 4:** *P*-value of distribution fit^a^^,^^b^ and optimal number of clusters.

		**Gaussian**	**Exponential**	**Log-normal**	**Clusters = 1**	**Clusters = 2**	**Clusters = 3**
Block 1	Avg	0.37	0.04	0.41			
	(SD)	(0.31)	(0.10)	(0.27)			
	% *p* > 0.1	62.5%	12.5%	87.5%			
	% Optimal^c^				62.5%	25%	12.5%
Block 2	Avg	0.30	0.06	0.57			
	(SD)	(0.26)	(0.10)	(0.30)			
	% *p* > 0.1	68.75%	18.75%	93.75%			
	% Optimal^c^				68.75%	12.5%	18.75%
Block 3	Avg	0.30	0.11	0.56			
	(SD)	(0.27)	(0.17)	(0.30)			
	% *p* > 0.1	62.5%	31.25%	93.75%			
	% Optimal^c^				87.5%	6.25%	6.25%

a *p-value > 0.1 indicates good fit*.

b *Distribution fit determined from the average center of drop locations in a given participant block*.

c *Optimality defined as the number of clusters (1, 2, or 3) which results in the greatest reduction in variability of individual drop location distances from the cluster center*.

The center of the near-confederate co-actor and near-target cluster regions had (x, y) locations of (46.4 cm, 46.07 cm) and (66.95 cm, 58.93 cm) respectively. This corresponded to an average distance of 61.5 and 89.6 cm from the participants, respectively, and 19.8 and 50.7 cm from the confederate co-actor's position, respectively. It remains unclear whether these locations represent a comfort-mode location, either with respect to the participant or the confederate co-actor. Consistent with previous research on third–order motor planning, it is possible that the reason why the distances of the two release/pass locations are beyond the participants' comfort reach distance (i.e., correspond to E/A ratios of 1.18 and 1.72, respectively) is because the actors (consciously or unconsciously) are attempting to maximize the beginning state comfort of the confederate co-actor (i.e., Gonzalez et al., 2011; Ray and Welsh, 2011; Meyer et al., 2013). For the current task, however, determining what constitutes the comfort-mode location or location of least-energy expenditure for the confederate co-actor is non-obvious and likely corresponds to a manifold of possible release/pass locations. Thus, it seems more likely that participants employed very little third–order motor planning from trial-to-trial and more or less picked a release/pass location very close to the confederate co-actor or within the reach of the confederate co-actor but closer to the target location. Thus, while participants tended to settle into one of two stable passing locations, it is unclear from the current experiment what about the participants or task-space drives the selection of a given pass location.

**Figure 4:**
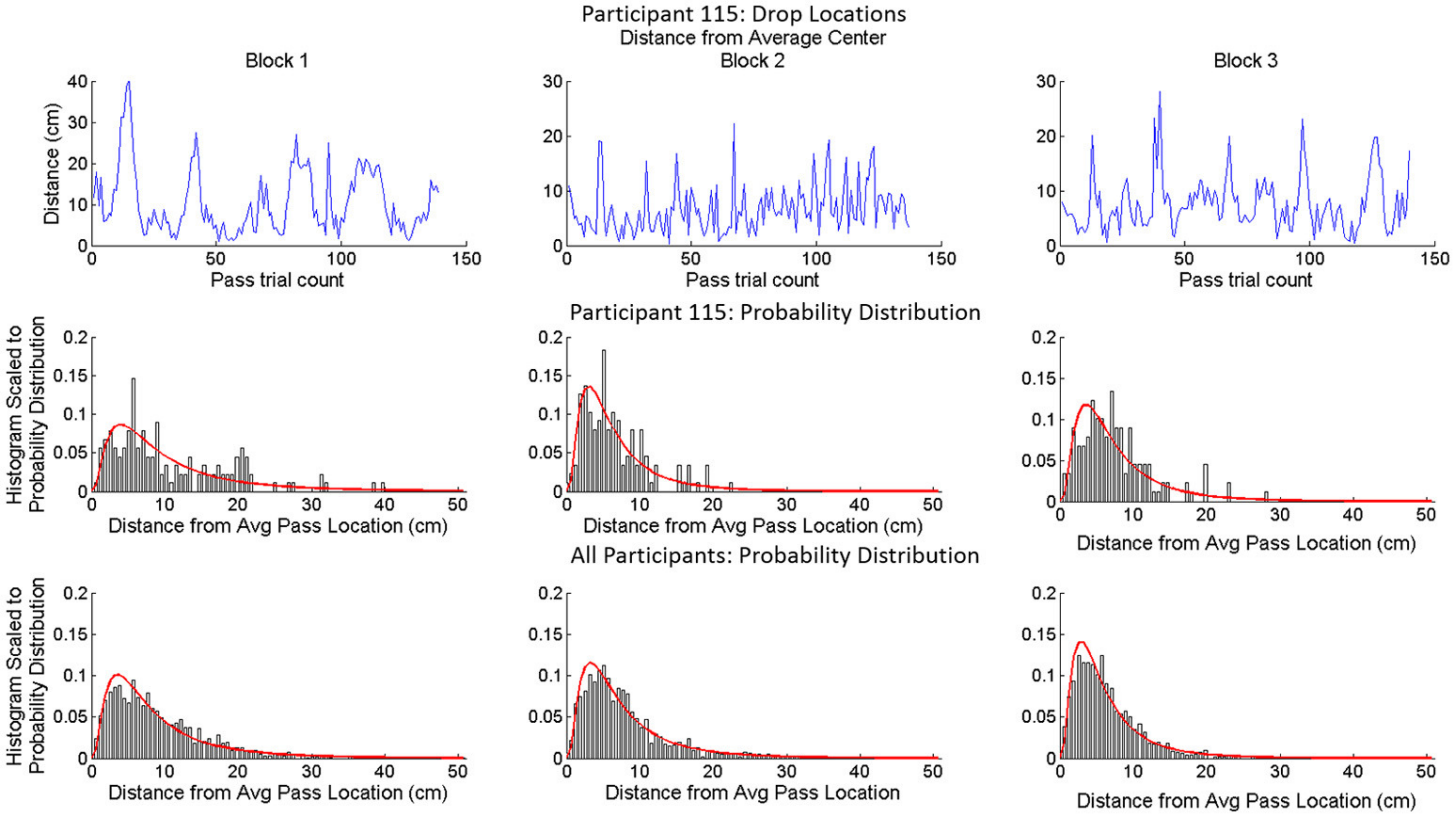
Example results from analysis of the distribution of pass locations around the average center of passes for a participant in a given condition. The top row is a time series plot of the distance from the average center on each passing trial. The middle row provides a histogram for each condition scaled to a log-normal curve (red line) fit to the data for that trial. The bottom row is the histogram for all participants in each of the conditions scaled to a log-normal curve fit to all of the participants' data for that condition.

Finally, in order to better understand the within cluster trial-to-trial pass/release location variability, that was not clearly accounted for by variation in target location or previous pass location, we classified the distribution of pass locations around the average center of pass locations for each participant and block. This was done by first calculating the squared Euclidean distance of each pass from the average center of all pass locations for each participant in a given condition. The probability distribution of this data was then estimated using a kernel density estimation and the probability distribution was fit to a Gaussian, Exponential and Log-normal distribution. A One-sample Kolmogorov-Smirnov test was used to determine the probability that the distribution of distances from the average center came from one of the possible sample distributions. Results of this analysis are displayed in Table 4 and illustrated in Figure 4. Consistent with recent research demonstrating how human behavioral variability over time exhibits significant degrees of persistence (e.g., Holden, 2002, 2005; Stephen and Mirman, 2010 for reviews), this analysis revealed that the distribution of pass locations around the average center tended to be log-normal (Table 4).

### How did the participants move?

To determine the trajectory dynamics of participant movement we separated the participant's pick and place movements into 3 sub-task movements: (1) object *pickup* movements or movements from the ready/start location to the object pickup location; (2) object *pass* movements or movements from object pickup to object release/pass; and (3) object *target* movements or movements from the object pickup to the object target drop-off location. The beginning and end of pickup and target movements corresponded to the first sample at which the center of the participant's hand-held motion sensor crossed the outer boundary of the corresponding start/object/target location. The beginning and end of pass movements corresponded to the first sample at which the center of the participant's hand-held motion sensor crossed the object pickup location (after picking up the object) and the moment the participant released the object for the confederate co-actor.

**Figure 5:**
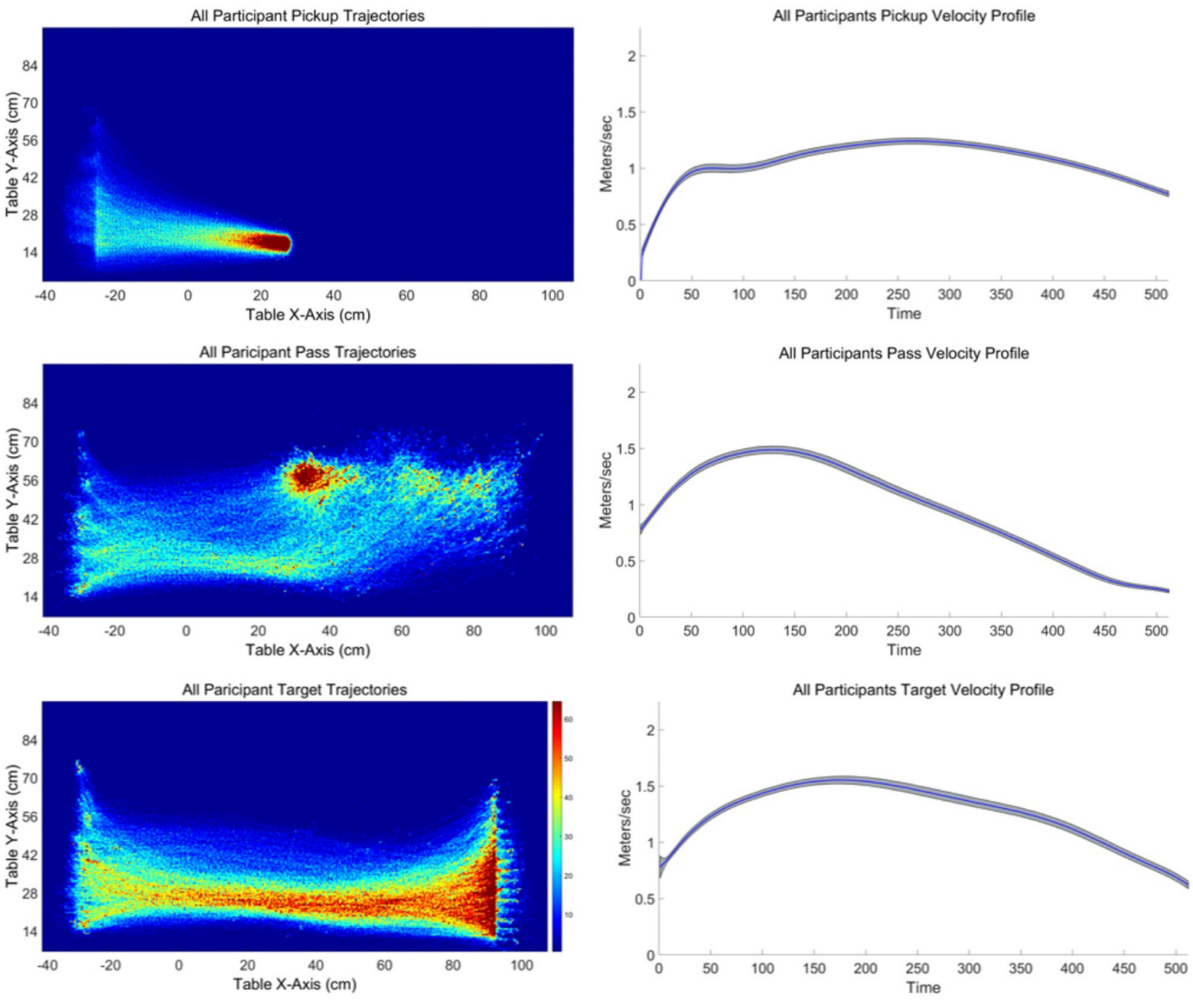
**(Left)** Heat maps illustrating all participant trajectories for the ready-pickup **(Top left)**, pickup-pass **(Left middle)**, and pickup-target **(Left bottom)** task-goal movements.

An illustration of the spatial trajectories observed for the different sub-task movements is provided in Figure 5 (left). These heat-map plots were created by dividing the table into 310 × 170 grid for pass and target trajectories and 930 × 510 grid for pickup trajectories due to the greater number of pickup trajectories. For each sub-task movement the number of times the participant's location was recorded in a given grid cell was recorded to create a histogram of trajectory locations in table coordinates. Colors are assigned to each cell from a color map with 64 colors. Overall, these heat-map plots revealed a consistent pattern of sub-task movement trajectories across participants. What is most apparent is that during pass and target movements participants consistently deviate from a straight-line path. More often than not, target and pass sub-task trajectories curved down toward the participant's standing position before curving back to the corresponding goal pass/release or target position. Although pickup movements trajectories were much closer to straight-line paths, there was also a consistent curve to the pickup movements for the closest and furthest pickup locations, albeit to a much lesser degree compared to pass and target sub-task movement curvature. Accordingly, the analysis of the sub-task movements focused on (a) the degree to which participants' total trajectories curved away from the shortest, straight line path between the start and end locations of the movement, (b) the deviation of the participants' initial heading or movement angle from the angle of the straight line path, and (c) the initial heading or movement angle (direction) of movement, as well as (d) the peak velocity and velocity profile of the sub-task movements. These trajectory measures were also important for determining whether the behavioral dynamics of these sub-task movements could be captured by an adapted version of the Fajen and Warren (2003, 2004) model described above.

The magnitude of movement curvature was quantified for each sub-task movement trajectory by calculating the area (m^2^) between the actual sub-task trajectory and the straight-line trajectory calculated from the first and last (*x, y*) location of the corresponding movement time-series. The area between the actual trajectory and straight-line trajectory was determined using the trapezoidal method of numerical integration. Prior to computing trajectory curvature, a spline interpolation procedure was employed to time-normalize the movement trajectories (to length of 512 points) in order to minimize variation in area estimations due to movement time variations. The initial movement or heading angle of each sub-task movement was calculated as the angle between the 1st and 9th points of the time-normalized movement trajectories. The angle (in degrees) was calculated with reference to the positive x-axis of the tabletop, such that horizontal straight-line movements directly across the tabletop from left to right would have an initial heading angle of 0° and horizontal straight-line movements directly across the tabletop from right to left would have an initial heading angle of 180°. The deviation from the straight-line angle was calculated as the initial participant movement angle minus the straight-line path angle, such that negative values corresponded to participant movement angles that were less than (under shot) the straight line path angle and positive values corresponded to participant movement angles that were greater than (over shot) the straight line path angle.

**Figure 6:**
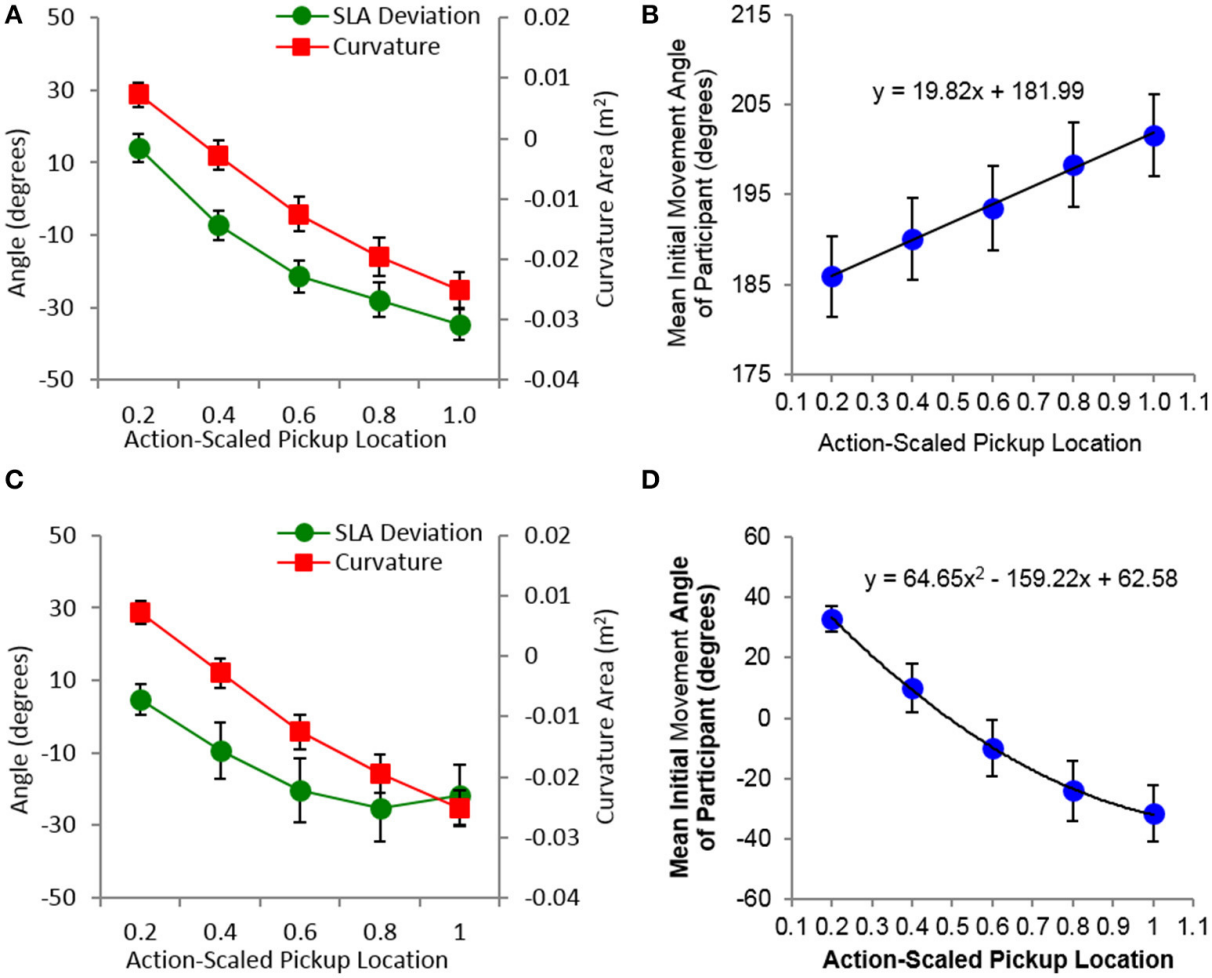
Mean trajectory curvature and deviation from straight-line angle (SLA) between the beginning and end point of a movement **(A,C)**, as well as an initial movement angle **(B,D)**, for pickup (top: **A,B**) and pass (bottom: **C,D**) sub-task movements as a function of the five action-scaled pickup locations. The black lines in **(B,D)** correspond to the best-fit line detailed in each plot. Error bars represent stand errors of the mean.

As can be seen from an inspection of Figure 6, the average degree of movement curvature for pickup and pass movements exhibited a somewhat linear change from positive to negative values as the action-scaled distance of pickup location increased, where positive curvature corresponded to movements that curved above the straight-line trajectory between the beginning and end locations of the movement and negative curvature corresponded to movements that curved below the straight-line trajectory between the beginning and end locations of the movement. Separate one-way repeated measures ANOVAs comparing the participant mean curvature values as a function of pickup location for pickup and pass sub-task movements revealed that this change was statistically significant [all *F*_(4, 60)_ > 120.97, *p* < *0.001*, $\eta_{p}^{2}$ > 0.90].

**Figure 7:**
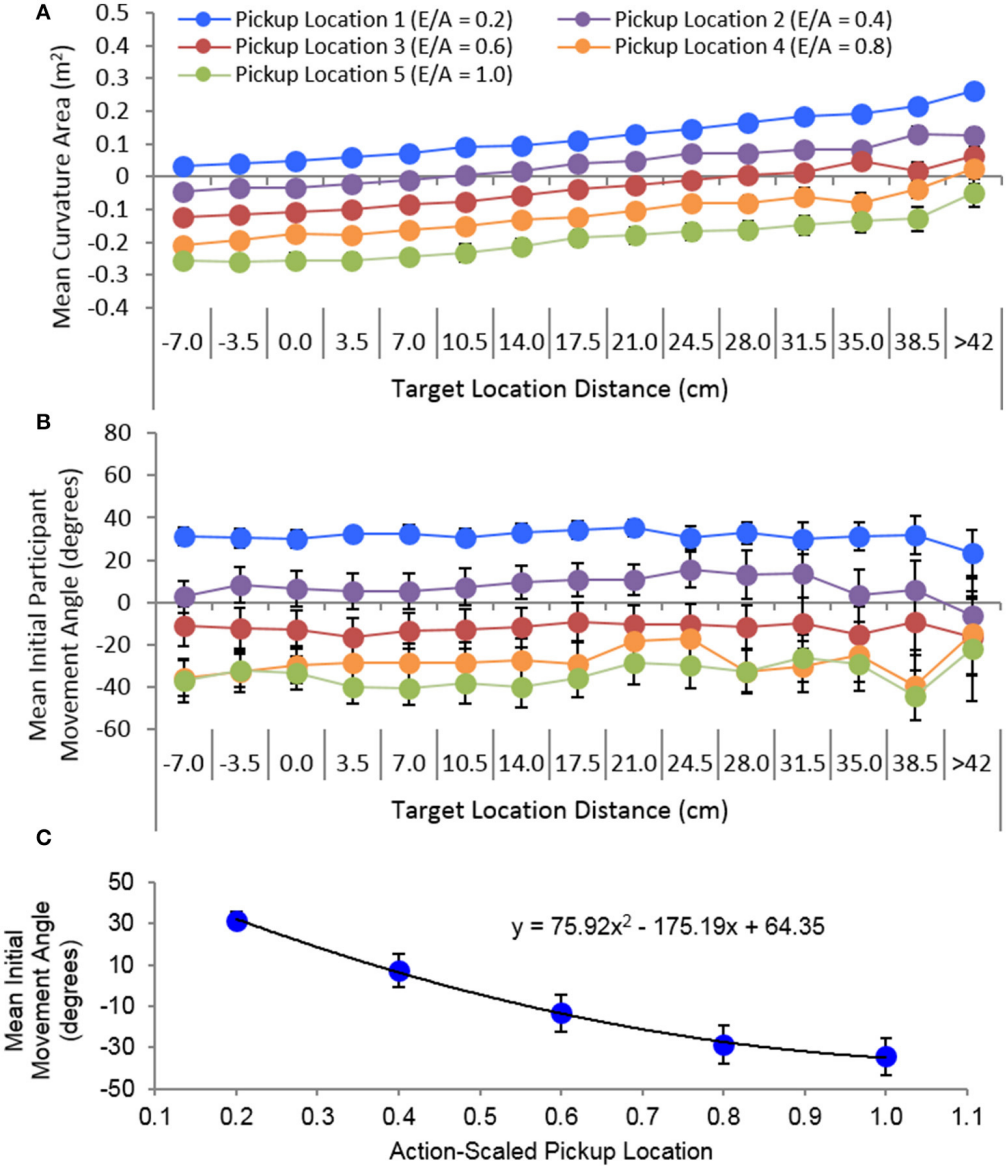
Mean trajectory curvature **(A)** and initial movement angle **(B)** average over participants for the target sub-task movements as a function of pickup and target location. Target location distance corresponds to the y-axis distance of the target location with respect to the start/ready hand location of participants. **(C)** Overall average initial movement angle as a function of the five action-scaled pickup locations. The black line in **(C)** corresponds to the best-fit line detailed in the plot. Error bars represent stand errors of the mean.

The data plotted in Figure 6 also indicates that degree and direction (positive vs. negative) of trajectory curvature for all sub-task movement types was directly related to the deviation of the initial movement angle from the straight-line angle between the beginning and end points of a movement. More importantly, although there was a change in initial movement angle as a function of the action-scaled pickup location for all sub-task movements [all *F*_(4, 60)_ > 25.54, *p* < 001 $\eta_{p}^{2}$ > 0.63], initial movement angle for the pass and target sub-task movements were largely independent of the end state distance or location of the movement. Specifically, for pass movements there was no significant difference between the participant mean initial movement angle for near-confederate co-actor and near-target participants, [*F*_(1, 12)_ = 2.24, *p* > 0.16, $\eta_{p}^{2}$ = 0.16]. Similarly, for target movements there was no change in participant mean initial movement angle as a function of target distance. This latter finding can be clearly discerned from inspection of Figure 7, where the overall mean initial movement angle is plotted for each pickup-target location combination for which target movements occurred. Taken together, this suggests that the trajectories exhibited by participants for each sub-task movement type were a result of participants employing a fixed, non-straight-line initial movement angle for each pickup location.

The highly predictable relationship between pickup location and initial movement angle for each sub-task movement type is illustrated in Figures 6B,D, 7C. For pickup movements this relationship was linear, with the range or change in the overall mean initial movement angle (185.88°–201.59°) much smaller than the range of mean straight-line angles (171.83°–236.15°) between the start/ready location and the five action scaled pickup locations. Again, this accounts for the positive to negative degrees in movement curvature as the pickup distance increased (see Figure 7A and Right-top panel of Figure 6). For the pass and target sub-task movements, the relationship between the overall mean initial movement angle was nonlinear, with the magnitude of change in initial movement angle decreasing as the distance of the pickup location increased. In addition, the initial movement angles employed when moving away from each pickup location were nearly exactly the same for the pass (range: 31.21° to −35.15°) and target movements (range: 31.35° to −33.89°), further emphasizing the fact that for the current task the intended end-point location played, on average, very little role in determining the initial movement angle when moving the object away from the pickup location. From the current study it is not clear what accounts for the observed initial trajectory angles. One possibility is that the observed initial angle ranges are the result of biomechanical constraints imposed on participant movements while reaching across the table.

Finally, the velocity of each sub-task movement was calculated from the non-normalized trajectory time-series. The resulting velocity time-series were then time normalized using the same 512 point spline interpolation procedure defined above. The overall average time-normalized velocity profiles for each sub-task movement are displayed in Figure 5 (right). As expected, participants exhibited non-constant, positively skewed velocity profiles for all sub-task movement types. There was no meaningful effect of pickup, release/pass, or target location with minimal variation in peak velocity across sub-task movements: pickup (Mdn = 1.473, Q1 = 1.43, Q3 = 1.494), pass (Mdn = 1.757, Q1 = 1.731, Q3 = 1.76); target (Mdn = 1.798, Q1 = 1.758, Q3 = 1.833). However, a Greenhouse-Geisser corrected one-way ANOVA did revealed a significant difference in peak velocity between the sub-task movements, [*F*_(1.187, 1.039)_ = 10.013, *p* = 0.004], with Bonferroni post-hoc analysis revealing that the peak velocity for the shorter distance pickup movements was significantly lower (*M* = 1.46 m/s, *SD* = 0.04 m/s) compared to the pass (*M* = 1.75 m/s, *SD* = 0.32 m/s) and target (*M* = 1.8 m/s, *SD* = 0.31 m/s) sub-task movements (both *p* < 0.025). There was no significant difference in peak velocity between the pass and target sub-task movements (*p* > 0.05).

## Modeling behavioral dynamics

The current study had two overall aims. The first aim was to identify the behavioral dynamics that underlie a relatively simple object pick and place task, in which one participant had to move objects from one tabletop location to another either alone or by passing the object to another co-actor. Of particular interest was how the changes in relative distance of the starting (appearance) and target goal (drop-off) locations of the to-be-moved objects with respect to a participant's standing position would influence (1) the affordance dynamics that characterized an actor's choice to move an object alone or to pass it to a confederate co-actor, (2) the location that a participant would choose to release an object when passing it to the confederate co-actor, and (3) the trajectory dynamics of the participant's hand movements when moving toward, with, or passing an object.

With regard to the affordance dynamics that characterized a participant's choice to move an object alone or to pass it to a confederate co-actor, results revealed that the participant's decision to pass or not-pass an object was a function of the intended target distance, with participants exhibiting a nonlinear phase transition between passing and not-passing at an average E/A ratio of 0.82 (i.e., ratio of y-distance of target/comfort reach distance of participant). Moreover, participants exhibited hysteresis, transitioning at a higher E/A ratio when target distance was increasing over trials compared to when target distance was decreasing over trials (i.e., 0.85 and 0.80 respectively), implying that the dynamics underlying this affordance actualization process were not only nonlinear, but were also multi-stable. Interestingly, although each participant was somewhat consistent with regard to the location that they chose to release/pass the objects to the confederate co-actor during passing events, the specific location chosen did not appear to be too dependent on the pickup location of the objects, nor the end target location. Rather, it appeared that participants either picked a location relatively close to the confederate co-actor's hand or relatively closer to the drop-off target locations and simply continued to release/pass objects in that same general location over the course of a trial block. Finally, participants exhibited a consistent pattern of curved movement trajectories across pickup, pass, and target movements, with movement curative a result of participants employing a stable set of non-straight-line initial movement angles that co-varied with pickup location. In addition, participants exhibited non-stationary velocity profiles, with peak velocity occurring within the first ½ of a corresponding pickup, pass, or target movement.

The second aim of the current study was to determine whether a simple behavioral dynamics model could be employed to capture these dynamics. More specifically, we were interested in whether an adapted version of the Fajen and Warren (2003, 2004) behavioral dynamics model of human locomotory navigation to a stationary target goal could be employed to capture the pick and place movements investigated here. We anticipated that at least two extensions would be required: (i) a non-stationary velocity function would have to be employed when modeling the hand-movement trajectories of participants; and (ii) a nonlinear action selection process to define whether participants passed or not. Below, we detail a preliminary model that not only incorporates these extensions, but exhibits the same qualitative movement and affordance dynamics exhibited by participants.

### Hand-movement dynamics

To model the dynamics of the participant's hand movements during object pickup, pass and target movements, a task specific parameterization of Equation (1) was employed. More specifically, the heading direction or angle, φ_*A*_, of a participant's (from this point on referred to as “agent,” *A*) hand or end-effector during pickup, pass and target movements was defined by

(2) $${\ddot{\varphi }}_{A}=-b_{g}{\dot{\varphi }}_{A}-k_{g}\left(\varphi_{A}-\theta_{g}\right)(e^{-c_{1}d_{g}}+c_{2}),$$

where ${\dot{\varphi }}_{A}$, and ${\ddot{\varphi }}_{A}$, correspond to the velocity and acceleration of the agent's end-effector heading angle, respectively, and *b* and *k* are damping and spring/stiffness terms, such that $-b_{g}{\dot{\varphi }}_{A}$ acts as a friction force on turning rate, and the function −*k*_*g*_(φ_*A*_ − θ_*g*_) operates to minimize the difference between the current heading angle, *f*_*A*_, and the angle θ_*g*_, of the corresponding sub-task goal/target location (i.e., the pickup location for pickup movements, the release/pass location for passing movements, and the target/drop-off location for target movements). A novel feature of Equation (2) is the presence of the factor (e^−*c*_1_*d_g_*^ + *c*_2_) in the second addend of the right-hand side. This factor modulates the effect of the term in Equation (2) operating to minimize the distance between the heading angle and the target angle. Specifically, it introduces an exponentially decaying function characterized by a constant offset parameter c_2_ and an exponential decay rate which is a function of the constant parameter c_1_ and the function

(3) $$d_{g}={\left[{\left(X_{g}-x_{A}\right)}^{2}+{\left(Y_{g}-y_{A}\right)}^{2}\right]}^{1/2},$$

where (*X*_*g*_, *Y*_*g*_) and (*x*_*A*_, *y*_*A*_) are the coordinates of the current sub-task goal location and the current location of the agent's end-effector (hand), respectively (see Fajen and Warren, 2004; for more details). The parameter *c*_2_ simply ensures that the rate of change in heading direction never goes to zero (Fajen and Warren, 2004).

It is important to appreciate that θ_*g*_ and *d*_*g*_ (defined in Equation 3), change as the position of the agent's hand/end-effector changes and are defined by

(4) $$\theta_{g}=\cos^{-1}\left[\frac{\left(Y_{g}-y_{A}\right)}{d_{g}}\right],$$

Now, recasting Equation (2) as a system of first-order differential equations and adding two extra equations defining the change in the (*x*_*A*_, *y*_*A*_) position of the agent's end-effector over time results in the following system of equations,

(5) $$\begin{array}{l} {\dot{z}}_{1}=z_{2}={\dot{\varphi }}_{A}\\ {\dot{z}}_{2}={\ddot{z}}_{1}={\ddot{\varphi }}_{A}=-b_{g}z_{2}-k_{g}\left(z_{1}-\theta_{g}\right)(e^{-c_{1}d_{g}}+c_{2})\\ {\dot{z}}_{3}={\dot{x}}_{A}=v_{A}\text{ sin }z_{1}\\ {\dot{z}}_{4}={\dot{y}}_{A}=v_{A}\;\cos\;z_{1}, \end{array}$$

where *v*_*A*_ is the movement velocity of the agent's end-effector (hand). In order for the model to capture the non-constant velocity profile observed in participants *v*_*A*_ is defined by means of the additional 2nd order differential equation

(6) $${\ddot{v}}_{A}=-b_{v}{\dot{v}}_{A}-k_{v}\left(v_{A}-C_{v}(1-e^{-d_{g}})\right),$$

where *b*_*v*_ and *k*_*v*_ operate as damping and stiffness terms on the rate of change of *v*_*A*_, which increases and decreases as a function of the target (goal) distance, *d*_*g*_. When the agent's end-effector or hand is far away from the target location, (1 − *e*^−*d_g_*^) approaches 1 and *v*_*A*_ increases. As the distance to the goal location decreases, however, (1 − *e*^−*d_g_*^) begins to approach zero and *v*_*A*_ decreases accordingly. *C*_*v*_ is a constant parameter that specifies the maximum velocity in m/s, such that the same equation can be used for a wide range of different movement distances, with differential peak velocities resulting for shorter and longer distances. Combining Equations (6) and (7) into a system of first order differential equations results in the end-effector (hand) movements or trajectories of an agent begin captured by

(7) $$\begin{array}{l} {\dot{z}}_{1}=z_{2}={\dot{\varphi }}_{A}\\ {\dot{z}}_{2}={\ddot{z}}_{1}={\ddot{\varphi }}_{A}=-b_{g}z_{2}-k_{g}\left(z_{1}-\theta_{g}\right)(e^{-c_{1}d_{g}}+c_{2})\\ {\dot{z}}_{3}={\dot{x}}_{A}=z_{5}\;\text{ sin}z_{1}\\ {\dot{z}}_{4}={\dot{y}}_{A}=z_{6}\cos z_{1},\\ {\dot{z}}_{5}=z_{6}={\dot{v}}_{A}\\ {\dot{z}}_{6}=-b_{v}z_{6}-k_{v}\left(z_{5}-C_{v}(1-e^{-d_{g}})\right), \end{array}$$

### Action selection dynamics

The dynamics of action selection observed in the current experiment were modeling using the equation

(8) $$\dot{x}=-\alpha +x-x^{3}$$

where *x* represents the state variable for action section (i.e., affordance mode) and α corresponds to the re-normalized E/A ratio calculated as

(9) $$\alpha =\left(\sigma -\frac{d_{g}}{R_{A}}\right)\delta$$

**Figure 8:**
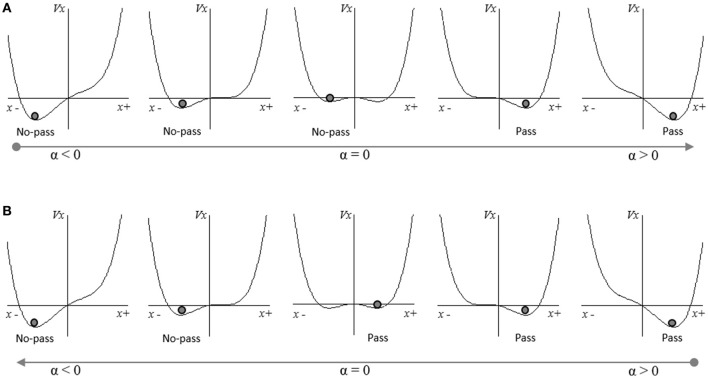
Illustrations of the potential function plots for Equation (8) for changes in the value of α. In **(A)**, the value of α increases from α < 0 to >0. As α approaches 0, the system becomes bi-stable but continues to converge on a stable solution at −*x*_*st*_. As α increases and −*x*_*st*_ becomes less stable the system eventually converges on the solution at +*x*_*st*_. In **(B)** the value of α decrease from α > 0 to α < 0, exhibiting the same characteristics as illustrated in **(A)** but in the opposite direction.

Here, *d*_*g*_ is the distance of the agent's end-effector (hand) to the target location, *R*_*A*_ is a measure of the agent's maximal preferred reach. α is the E/A ratio participants typically switch between behavioral modes, and σ and δ are constant scaling factors. As can be seen from an inspection of Figure 8, where Equation (8) is plotted as the potential function

(10) $$V\left(x\right)=\;\alpha x-\frac{x^{2}}{2}-\frac{x^{4}}{4}$$

this system results in a saddle-node bifurcation as α is scaled up or down past ±α_*c*_ (approximately ±α_*c*_ = 0.35). Moreover, the system exhibits a region of bi-stability between ±α_*c*_ and corresponding hysteretic behavior. More specifically, for α < −α_*c*_ and α>+α_*c*_ the system has a single stable fixed point at −*x*_*st*_ and +*x*_*st*_, respectively. For −α_*c*_ < α < +α_*c*_, however, the system has two stable fixed points at, −*x*_*st*_ and +*x*_*st*_, respectively, as well as an unstable fixed point between the two. This system has previously been employed to capture the nonlinear transitions in categorical speech perception (Tuller et al., 1994; Tuller, 2005), attitude change (Richardson et al., 2014) and conciliation dynamics during conflict situations (Coleman et al., 2007), and appears to represent a generic nonlinear decision or action selection process (van Rooij et al., 2013). For the current pick and place task, we arbitrarily defined convergence on a stable fixed point at −*x*_*st*_ to specify non-passing (i.e. moving alone) and convergence on a stable fixed point at +*x*_*st*_ to specify passing. Accordingly, when α < −α_*c*_ and α > +α_*c*_ the system is mono-stable and the agent always converges on the one stable corresponding action mode. However, when −α_*c*_ < α < +α_*c*_ the action selection dynamics are bistable, with the likelihood of converging on one of the two corresponding action modes (i.e., passing or not-passing) a function of the relative stability of the two fixed points and the previous state of system.

## Model simulation

**Figure 9:**
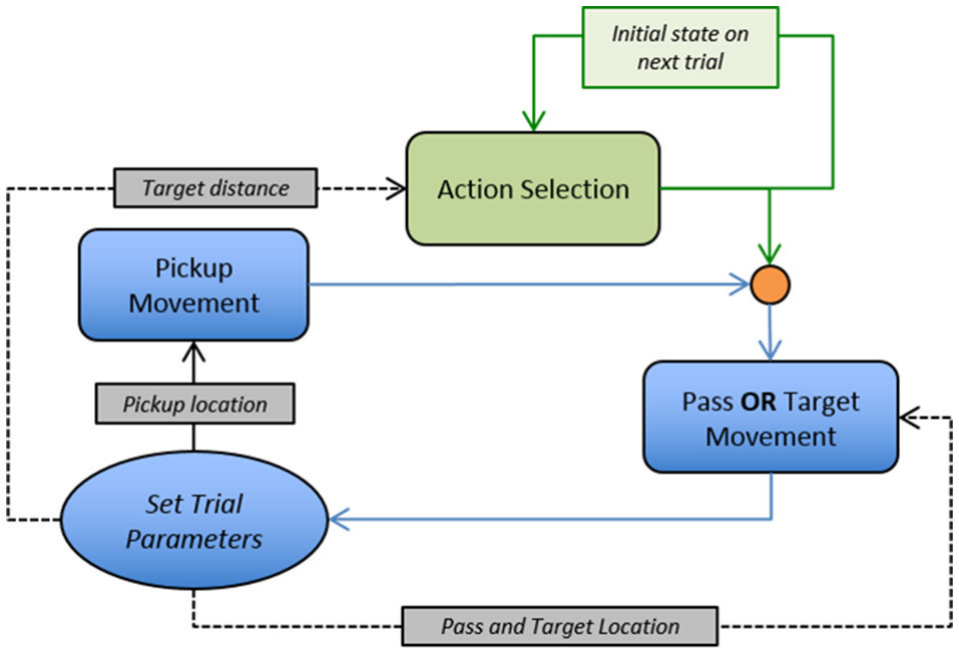
Flow diagram illustrating the implementation of the behavioral dynamics model for data simulations. Movement trajectory dynamics for the pickup, pass, and target movements defined by Equation (7). Action selection dynamics defined by Equation (8). Note, that the orange circle is a Heaviside function that defines the goal location as either the pass location *or* the target location depending on the output of the action selection dynamics.

To determine whether the movement trajectory dynamics defined by Equation (7) and the action selection dynamics defined by Equation (8) were able to qualitatively capture the behavioral dynamics exhibited by participants in the current pick and place task, a MATLAB (2014a) simulation was conducted. A flow diagram illustrating the structure of the simulation is provided in Figure 9. The simulated environment consisted of a 1.50 × 0.89 meter rectangular space matching the experimental table's dimensions. Pickup locations were calculated based on the average participant comfort reach distance of 52.2 cm. The initial model and simulation target locations matched the ready and target locations in the original task setup. Eight different simulations sequences were conducted, with each simulation sequence consisting of 3 blocks (ordered, random, ordered) of 200 trials (600 trails in total for each simulation sequence). For four of the simulations the passing location corresponded to the near-target passing location (0.7695, 0.5893) observed in the experimental data. For the other four simulations the passing location corresponded to the overall average near-confederate co-actor passing location (0.464, 0.5607) observed in the experimental data. Experimentally observed pass location variability is likely due to the many complex interactions from which this passing behavior emerges (Holden, 2002, 2005; Stephen and Mirman, 2010). However, in our model this variability is simulated using a sequence of random values generated from a lognormal distribution that were added to the passing location in order to produce a pass location distribution that was similar to the original data.

The action selection dynamics (Equation 8) were integrated for 1,500 steps using the MATLAB ODE45 function with the end state of the integration used to drive the decision to pass or go to the target. The output state of the action selection equation was stored as an input for integration of the action selection equation in the next trial (*x* = 0 for the first trial in a sequence). Based on the results of the original experiment, the initial trajectory angles for each sub-task movement type for each trial was calculated using the regression equations in Figures 6, 7 for pickup and pass/target movements, respectively. Random noise was added to the initial angle from a uniform distribution with min/max values of ± 20°. The movement dynamics (Equation 7) were integrated separately for each sub-task movement using the Euler integration (0.01 time step), with integration terminated when the model location was within 4 cm of the target location. Random noise was added to the model heading direction, φ_*A*_, at each time step of the integration using a uniform distribution with min/max values of ± 1.14°.

**Figure 10:**
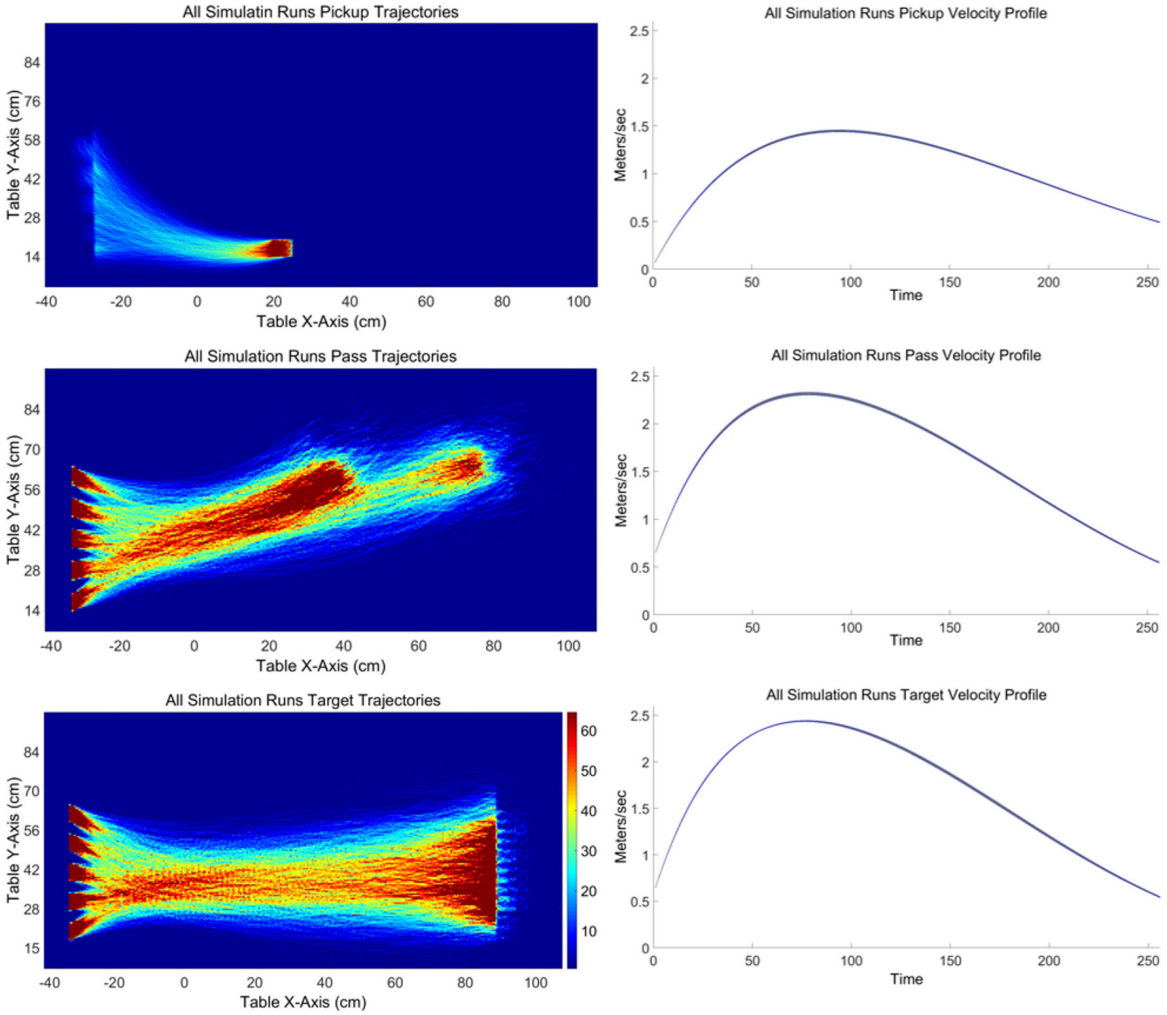
**(Left)** Heat maps illustrating all simulation run trajectories for the pickup **(left top)**, pass **(left middle)**, and target **(left bottom)** sub-task goal movements. **(Right)** Average simulation velocity profile for each sub-goal trajectory, ready-pickup **(right top)**, pickup-pass **(right bottom)**, and pickup-target **(right bottom)**, in meters per second with 5% and 95% confidence intervals indicated in gray.

Heat-maps were created using the same method as in the original experiment; however, due to the reduced variation in the model a 1,240 × 680 grid was used for pickup trajectories. As can be seen from an inspection of Figure 10 the overall heat-map plots revealed patterns of sub-task movement trajectories similar to those observed in the original experiment. As observed in actual participants, the model deviates away from a straight-line path during pass and target movements with a trajectory that tended to curve down toward the bottom of the task space before curving back to the corresponding goal pass/release or target position. This curvature is driven in the model by the initial trajectory angle set at the beginning of each sub-task movement. When the initial angle is calculated using the straight-line angle between the initial trajectory location and the sub-task goal location, the model does not exhibit this curving behavior, even with noise added to the heading direction. This suggests that when participants pick up the object they immediately start toward the other side of the table but do not decide exactly where they are going until later in the trajectory. The observed curved trajectories emerge from the initial conditions of the sub-task trajectory and the dynamics of the system. Velocity also plays a role in the curvature of the trajectory, with trajectories tending to curve more and longer when the velocity is high. As can be seen in the heat-map of the passing trajectories, the curve toward the passing locations tends to be less abrupt in the simulations than observed in the original experiment. One possibility that might account for this difference may be that the decision to pass occurs at some point after pickup before the participant has located the target location. Future studies could look at factors that further affect trajectory curvature, including the possibility that action selection occurs online and not at a single point within a task-goal trajectory.

**Figure 11:**
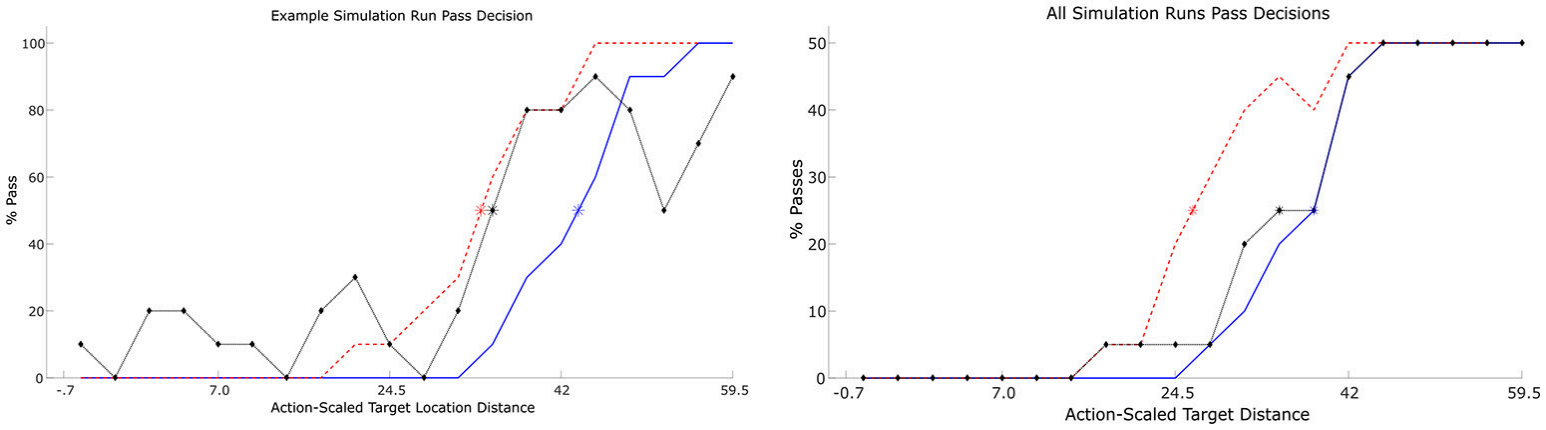
Percent passes for each target location (n = 20) for a single simulation run **(Left)** and for all 8 simulation runs **(Right)**. The red dashed line indicates the percentage of passes for each target as the targets are moving away from the participant. The blue solid line indicates the percentage of passes as the targets are moving toward participant. Random target appearance are represented by the black dotted line with handles. Asterisks represent the point at which 50% of decisions were passes and 50% were not (note that this point could occur between target locations). Each target location was presented 5 times each per Ascending and Descending conditions and 10 times for the Random condition.

Figure 11 illustrates the percentage of passes performed for each target location depending on the appearance order of the targets (ascending, descending, or random). As can be seen in Figure 11, the action selection dynamics of the model exhibit hysteresis similar to observations in the original experiment (see Figure 2). To verify that the hysteretic effect observed in the simulation experiment was significant, a one-way repeated measures ANOVA was conducted comparing the distance (target location) that the model switched between passing and not passing as a function of target location order (i.e., ascending, descending, and random) in each simulation run. This analysis revealed a significant effect of target location order, [*F*_(2, 12.007)_ = 13.946, *p* < 0.001, $\eta_{p}^{2}$ = 0.666], with Bonferroni *post hoc* analysis indicating that pass/no-pass transition distance for the ascending target order was significantly higher compared to the pass/no-pass transition distance for the descending target order (*p* = 0.005). There was no significant difference between either the ascending or descending and random target location orders (*p* > 0.05).

Finally, as can be seen in Figure 10 (right), the shape of the average velocity profile is qualitatively similar to the average velocity profile observed in the original experiment. The peak velocity occurs around the first 1/3rd of the trajectory, with a difference in the magnitude of average peak velocities between the pickup sub-task goal and the target and pass sub-task goals.

## Conclusion

The current study identified and modeled the affordance and nested sub-task movement dynamics of a simple pick and place task. As expected, the results revealed a consistent pattern of behavioral action across participants, with the transition between social (object passing) and solo action (not passing or moving the objects alone) determined by an intrinsic relation between the participant's action capabilities and the physical task-relevant constraints (Warren, 1984; Mark, 1987; Warren and Whang, 1987; Kinsella-Shaw et al., 1992; Richardson et al., 2007; Harrison et al., 2016). The hysteretic nature of the transition from solo- to social-action was also expected and provided further evidence that the perception and actualization of mutually destructive (and constructive) affordance possibilities is governed by nonlinear, multi-stable dynamical processes (Kelso, 1995; Frank et al., 2009; Richardson and Kallen, 2015). The verified implication of these findings was that a simple nonlinear bifurcation function (Equation 8), parameterized by a normalized E/A ratio of the participant's comfort reach capabilities relative to the distance of the intended object target goal location (Equation 9) could be employed to effectively capture the affordances dynamics exhibited by participants (see Figure 11).

Interestingly, participants consistently released/passed the object in roughly the same location throughout the experiment, either near the targets or near the co-actor. Although nearly all participants settled on one of these two pass location strategies, it remains unclear why any particular participant chose one passing location over the other and further research is needed to investigate how and why these location preferences emerged. It is significant, however, that the pass location chosen by a given participant was dependent on task-invariant features of the task-space, namely, the confederate co-actor's hand location or the confederate co-actor's hand location relative to the target locations. Together with the fact that a participant's chosen pass location was independent of changes in trial-to-trial object appearance and target distance locations, this suggests that participants chose their pass location with respect to the global structure of entire task context. This suggests that predictions about a participant's pass location can be made without reference to smaller scale fluctuations that occur as the task unfolds. Moreover, precise prediction about the specific release/pass location chosen by a given participant appears to be of little importance with regards to functional task completion or with regards to modeling the behavioral dynamics observed. That is, so long as an object is released/passed in a location that can be easily reached by the confederate co-actor, the object can be picked up and moved effectively by the co-actor. This is not to say that there are not locations that would result in more efficient or optimal patterns of behavior (and less overall energy expenditure); rather this appears to be less important than the predictability of current and future release/pass locations (Cakmak et al., 2011; Strabala et al., 2013). Indeed, the specification of a pass in the current task context was defined by the invariance of returning to the same chosen release/pass location, not the degree to which the release/pass location corresponds to some optimal pass location. Accordingly, the degree to which third–order motor planning (Ray and Welsh, 2011; Meyer et al., 2013) operated to constrain the behavior of participants appeared to be minimal in the current task.

The results of the current study also demonstrated how the trajectory dynamics of the participant's sub-task hand movements, including movement velocity, could be effectively captured by an adapted version of the Fajen and Warren (2003, 2004) behavioral dynamics model of locomotory path navigation. The significance of this finding is twofold. First, it highlights how the same low-dimensional behavioral dynamics can operate to constrain multiple (and often nested) levels of human activity. Second, it suggests that, with the exception of pass locations that require further investigation, knowledge of *what, when, where and how* to move or act during a social interaction is often lawfully defined by these low dimensional task dynamics and, thus, can emerge spontaneously and in real-time with little a priori planning. Indeed, participants in the current task did not appear to plan out their sub-task movement trajectories from the outset, nor did they even appear to plan their sub-task movement with regards to the shortest path of the final end state or task goal. In fact, participants did not adjust their initial angle to the specific sub-task goal location on a given trial, even when the location of the sub-task goal was predictable. Instead, participants essentially moved in the general direction of the next sub-task goal, shaping the needed trajectory over the course of movement. As a result, the movement trajectory and velocity profiles that occurred were simply an emergent product of historically dependent initial conditions (parameterizations) operating within a set of well-defined task constraints.

Clearly, the confederate co-actor in the current pick and place task played a minimal role. It is therefore possible that the observed dynamics would have been different if the confederate co-actor was more engaged in the task (e.g., picked up and passed objects also). In particular, when two or more agents are simultaneously active in a shared task space the decisions on whether to pass and where to pass are dependent on the behavioral movements and action possibilities of both actors together. Although future research is planned to investigate the behavioral dynamics of a more complex joint-action pick and place scenario, it is possible that very minimal changes to the current pick and place model will be required to capture the dynamics of such joint action behavior. That is, it seems likely that the movement trajectory dynamics of actors in a truly joint action pick and place task would be almost identical to those observed in the current task, with the only addition needed to Equation (2) being an obstacle avoidance coupling to prevent the actors bumping into each other. The action selection dynamics of the actors would also need to be coupled, such that the affordance dynamics of each actor are mutually dependent. However, these minimal changes are easily implemented and would *not* increase the dimensionality of the system of equations detailed above. Of major interest, would be whether such minimal changes could produce patterns of behavioral joint-action as complex as those that would be expected during real human-human behavior—i.e., the emergence of complexity from non-complexity.

Finally, the Fajen and Warren model of path navigation has been successfully implemented in robotic systems for local obstacle avoidance and path navigation in novel environments (Huang et al., 2006; Nemec and Lahajnar, 2009). Building on this previous work and the current research, a future next step is to explore the application of the proposed model in human-robot and human-virtual avatar joint-action pick and place tasks. Demonstrating how this and other task or behavioral dynamics models can be employed for the development of robust human-machine systems will not only further validate the effectiveness of the such models for effectively capture human multiagent behavior, but will also further emphasize the degree to which such models are able to provide a grounded explanation of multiagent behavior in general.

## Ethics statement

This study was carried out in accordance with the recommendations of the University of Cincinnati Institutional Review Board with written informed consent from all subjects. All subjects gave written informed consent in accordance with the Declaration of Helsinki. The protocol was approved by the University of Cincinnati Institutional Review Board.

## Author contributions

ML: Collected and analyzed data, developed, tested and parameterized model. RK: Contributed to experimental design as well as theory and model development. SH: Contributed to model development and testing. Contributed to data analysis and interpretation. MD: Contributed to model development (instrumental in development of velocity model). Contributed to data interpretation. AM: Contributed to experimental design. Contributed to data analysis and interpretation as well as model development and testing. MR: Contributed to model development, characterization, and testing. Contributed to data analysis, interpretation, and presentation. Contributed to experimental design.

### Conflict of interest statement

The authors declare that the research was conducted in the absence of any commercial or financial relationships that could be construed as a potential conflict of interest.

## Footnotes

^1^Fajen and Warren (2003) have modeled the change in φ with respect a stationary point-mass obstacle as by adding the function $+\sum_{i}^{N}k_{o}\left(\varphi -\theta_{oi}\right)e^{-|\varphi -\theta_{oi}|}f\left(d_{oi}\right)$ to Equation (1), where + *k*_*g*_(φ − θ_*o*_) operates push the agent's heading direction, φ, away from the heading angle, θ_*o*_, that leads toward the obstacle as a function of distance, *f*(*d*_*o*_). Here, the addition of the exponential function, ($e^{\left|-\varphi -\theta_{o}\right|}$) , ensures that the angular acceleration away from an obstacle quickly rises near the obstacle and results in a positive (right) truing rate when heading to the right of θ_*o*_ and a negative (left) truing rate when heading to the left of θ_*o*_.

^2^In particular, note that interpretation of pass receiver movements as playing either a communicative or anticipatory role in task performance requires some understanding of the task dynamics driving the passer's decisions and behaviors independent of those movements.

^3^Herarchical logistic regression analyses were also performed to confirm that target distance was the only task variable to significantly predict participant pass/no-pass behavior. Not only did this analysis further confirm that there was no correlation between participant trial-by-trail pass/no-pass decisions and object pick location, but it also verified that the variable interaction between pickup and target location did not predict participant trial-by-trail pass/no-pass behavior beyond that predicted by target distance alone.

^4^A participant-by-participant hierarchical logistic regression analysis with decision to pass on current trial as the dependent variable and the current target location and previous pass decision as an independent variable resulted in an average Nagelkerke's *R*^2^-value of .903 and .943 for the ascending a descending conditions, respectively (all χ^2^ > 200.00, *p* < 0.001).
